# Supplementary material for: In vivo characterization of Drosophila golgins reveals redundancy and plasticity of vesicle capture at the Golgi apparatus
Source: Curr Biol. 2022 Nov 7;32(21):4549–4564.e6. doi: 10.1016/j.cub.2022.08.054 (PMC9849145; doi:10.1016/j.cub.2022.08.054)
Supplement: Document S1. Figures S1–S7 [file mmc1.pdf]

Current Biology, Volume *32*

Supplemental Information

In vivo characterization of *Drosophila* golgins reveals redundancy and plasticity of vesicle capture at the Golgi apparatus

Sung Yun Park, Nadine Muschalik, Jessica Chadwick, and Sean Munro

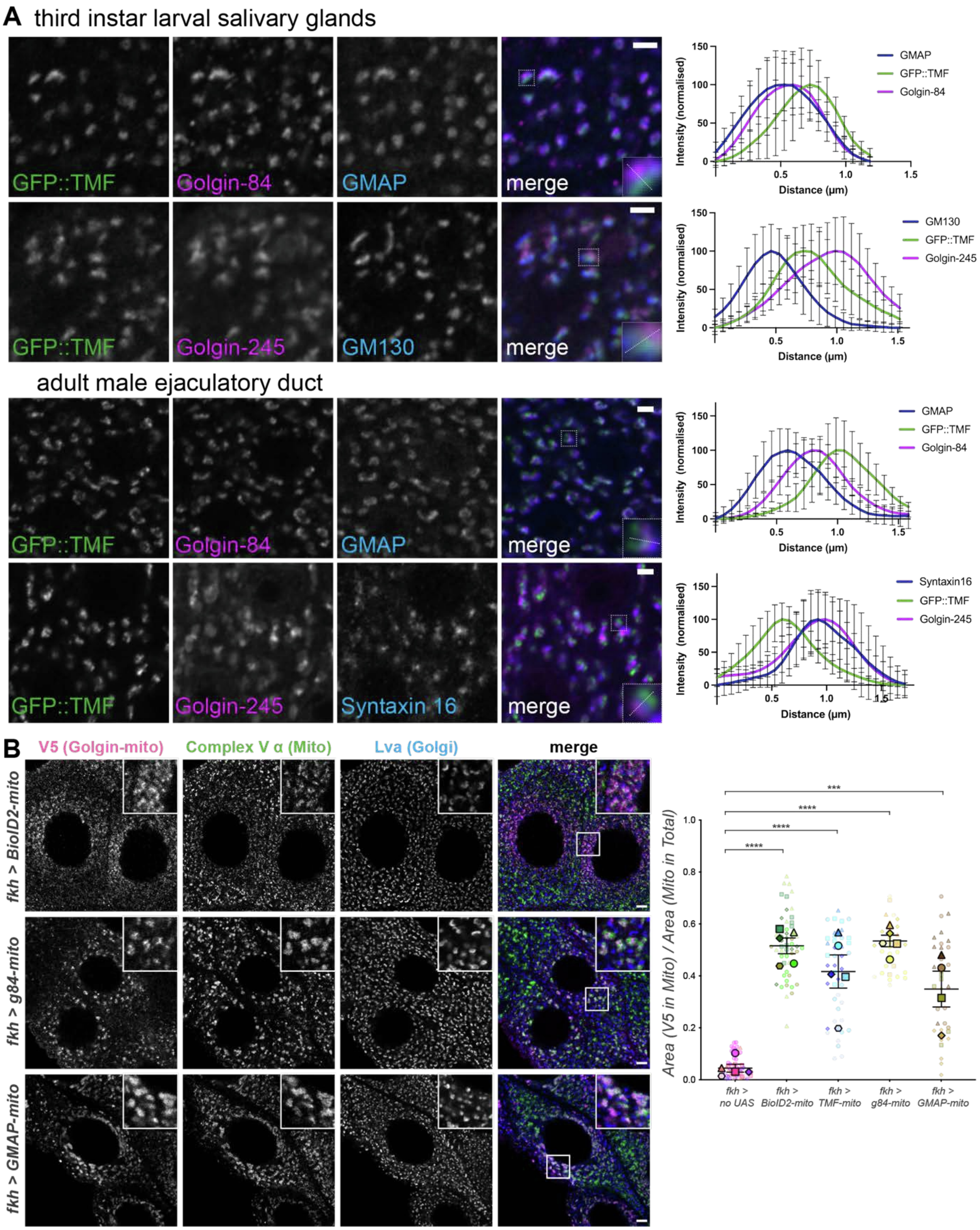

Figure S1. Localisation of GFP::TMF, and mitochondrial relocation of Golgin-84 and GMAP in *Drosophila*. Related to Figure 1.

(A) Confocal micrographs comparing GFP::TMF to the indicated golgins in salivary glands and the male ejaculatory duct. The insets show a higher magnification of the Golgi highlighted in the image. The dotted

line illustrates a typical line profile used for quantification, with the graphs showing the normalised means of 10 line profiles of the indicated proteins across the Golgi stack (errors bars, SD). GFP::TMF localises trans to GMAP (cis Golgi) and Golgin-84 (early rims). GFP::TMF localises in between GM130 (cis) and Golgin-245 (trans). GFP::TMF localises cis to Golgin-245 and Syntaxin 16 (both trans Golgi). Scale bars: 2µm. Full data in Data S2.

(B) Confocal micrographs of L3 salivary glands labelled for the V5 epitope tag in the indicated golgin-mito (magenta), and also mitochondrial Complex V α (green) and Golgi-marker Lava lamp (Lva, blue). Insets show zoom-in views of the boxed regions in the merge. Also shown is a negative control (BioID2-mito). Scale bar: 5 µm. Quantification of combined golgin-mito relocation data from Figure 1C and S1A. Degree of relocation calculated by ratio between area of V5 binaries in mitochondria and total area of mitochondria. Cell-by-cell ratios plotted as small partially transparent points. Larval ratios (averaged cell-by-cell ratios) plotted as large opaque points. Points are shaped and coloured according to larval replicate, with the coding scheme of cell-by-cell points matching that of their corresponding larval points. The mean larval ratio (line) and SEM (bars) are plotted for each genotype. Data analysed by one-way nested ANOVA using Tukey's multiple comparisons, *** = $p \leq 0.001$, **** = $p \leq 0.0001$. n = 5 larvae (4 for GMAP-mito), with 4-15 cells segmented per larvae. Full data in Data S1.

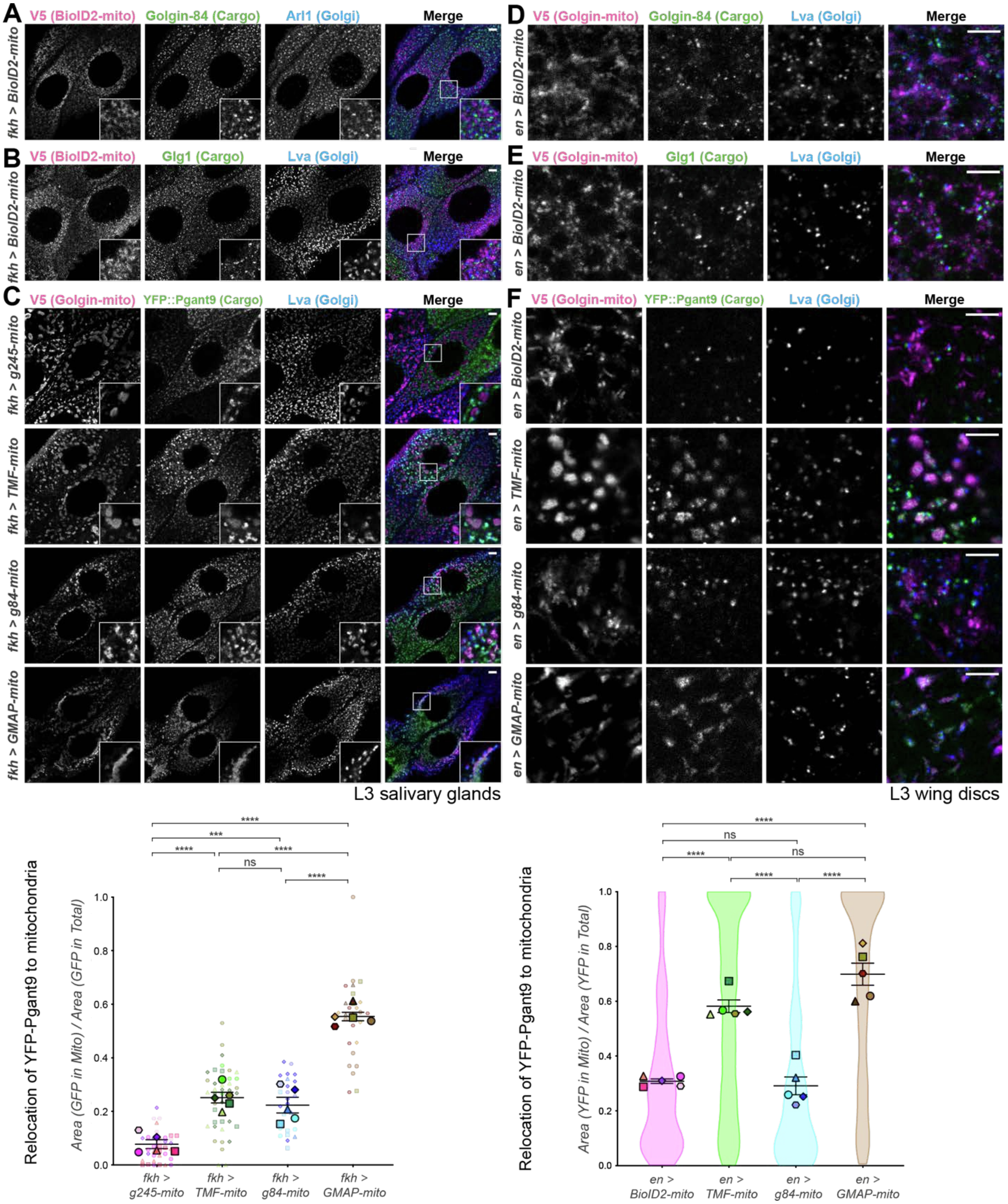

Figure S2. Capture of YFP::Pgant9 containing vesicles by intra-Golgi golgin-mitos. Related to Figures 2 and 3.

(A) Confocal micrographs of L3 salivary glands expressing the BioID2-mito negative control, and labelled for V5 (magenta), endogenous Golgin-84 (green), and Golgi-marker Arl1 (blue).

(B) As (A) except the labelling was for V5 (magenta), endogenous Glg1 (green), and *trans*-Golgi marker Lva (blue). Scale bars: 5 μm.
(C) Confocal micrographs of L3 larval salivary glands expressing the indicated V5-tagged golgin-mito constructs and labelled for V5 (magenta), endogenous YFP::Pgant9, and Golgi-marker Lva (blue). Endosome-to-Golgi vesicle tether Golgin-245-mito was used as a negative control. Insets show zoom-in views of the boxed regions in the merge. Quantification of YFP::Pgant9 relocation uses ratios between cargo in mitochondria and total area of cargo. Plots show cell-by-cell ratios plotted as small partially transparent points. Larval ratios (averaged cell-by-cell ratios) plotted as large opaque points. Points are shaped and coloured according to larval replicate, with the coding scheme of cell-by-cell points matching that of their corresponding larval points. The mean larval ratio (line) and SEM (bars) are plotted for each genotype. Data analysed by one-way nested ANOVA using Tukey's multiple comparisons, ns = not significant, ** = $p \leq 0.01$, *** = $p \leq 0.001$, **** = $p \leq 0.0001$. n = 5 larvae for each genotype, with 3-12 Pgant9-positive cells segmented per larvae. Full data in Data S1.
(D) Confocal micrographs of L3 wing discs expressing the BioID2-mito negative control, and labelled for V5 (magenta), endogenous Golgin-84 (green), and Golgi-marker Arl1 (blue).
(E) As (A) except the labelling was for V5 (magenta), endogenous Glg1 (green), and *trans*-Golgi marker Lva (blue). Scale bars: 5 μm.
(F) Confocal micrographs of L3 larval wing discs expressing the indicated golgin-mito constructs and labelled for V5 (magenta), endogenous YFP::Pgant9 (green), and Golgi-marker Lva (blue) in L3 larval wing imaginal disc. YFP::Pgant9 expression was restricted to small patches throughout the wing disc, and so the wing pouch cells imaged for other markers were not imaged, but rather a patch of notum cells, as these cells were one of the few areas where YFP::Pgant9 and *engrailed* expression intersects. Insets show zoom-in views of the boxed regions in the merge. Scale bars: 5 μm. Quantification of YFP::Pgant9 relocation experiment uses the ratio between area of cargo in mitochondria to total area of cargo. For each quantification, cell-by-cell ratios from each larval replicate were pooled for each genotype and represented as truncated violin plots. Disc ratios (averaged cell-by-cell ratios) were plotted as large opaque points. Points are shaped and coloured according to larval replicate, with the coding scheme of cell-by-cell points matching that of their corresponding disc points. The mean larval ratio (line) and SEM (bars) are plotted for each genotype. Data analysed by one-way nested ANOVA using Tukey's multiple comparisons, ns = not significant, * = $p \leq 0.05$, ** = $p \leq 0.01$, *** = $p \leq 0.001$, **** = $p \leq 0.0001$. n = 5 discs for each genotype, with typically 20-55 Pgant9-positive cells segmented per disc. Full data in Data S1.

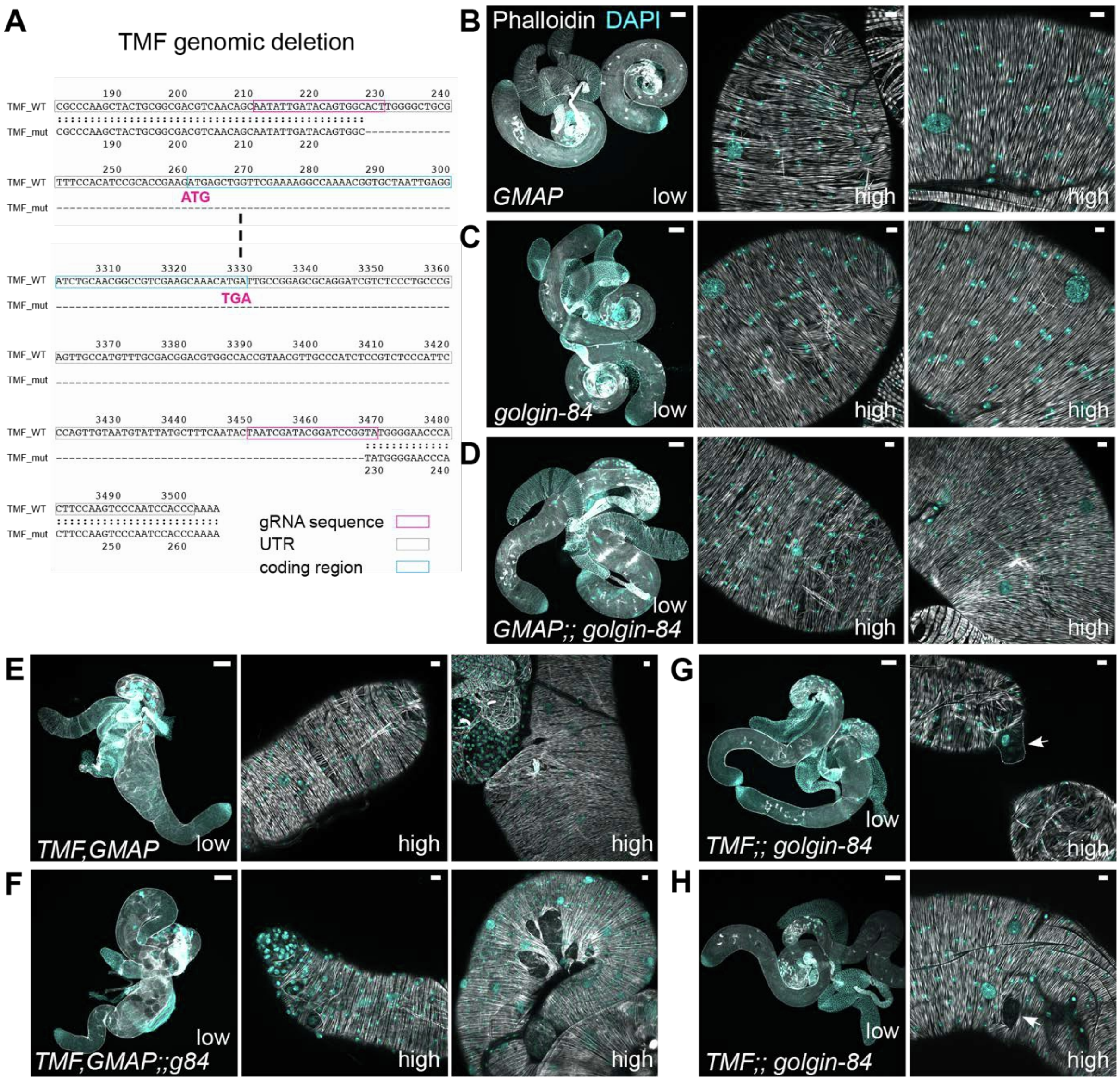

Figure S3. Testis muscles are not affected in *GMAP* and *golgin-84* single and double mutants. Related to Figures 4 and 5.

(A) Pairwise sequence alignment of the *TMF* wild-type genomic sequence versus that obtained for a PCR product amplified from the *TMF* deletion mutant. Two gRNAs were used, and this has resulted in the removal of the entire coding region.

(B-H) Confocal micrographs of F-actin (phalloidin, grey scale) and DNA (DAPI, cyan) in testes of the indicated golgin mutants. Images were taken at low magnification to show the whole testes (low), or higher magnification to show the actin in the muscle sheath (high). *GMAP* and *golgin-84* single and double mutants do not show any muscle defects (B-D). Holes (E, F), and lack of muscles at the tip of the testis (F), are the weaker phenotypes observed in *TMF, GMAP* and *TMF, GMAP;; golgin-84* mutants. *TMF;;golgin-84* double mutants (G,H) occasionally lack muscles at the tip of the testis (see dotted line

and arrow in G) and display holes (see arrow in H). The phenotype is similar to that seen in *TMF* single mutants. Scale bars: low: 100 µm, high: 10 µm.

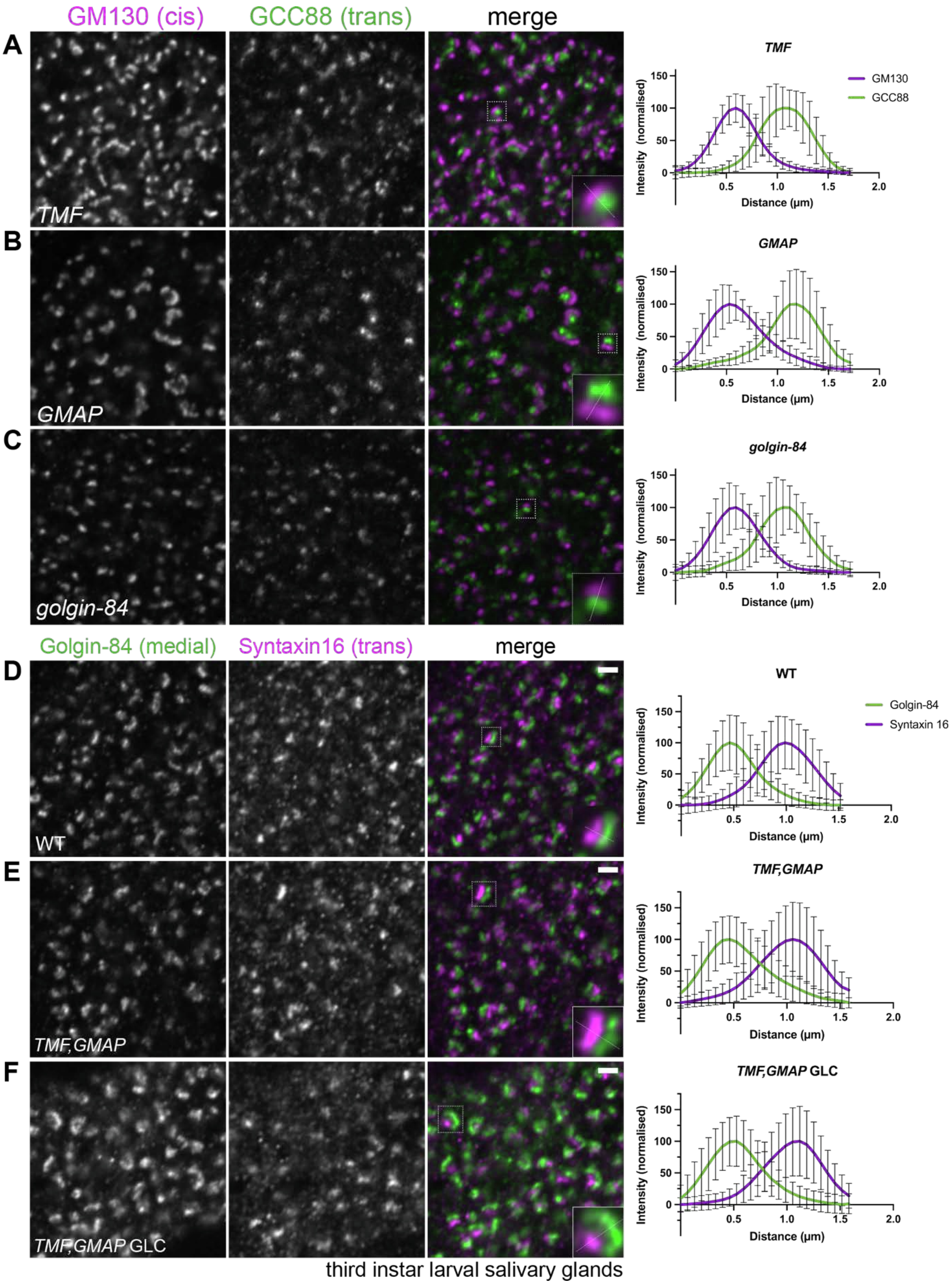

Figure S4. Intra-Golgi golgin mutants retain Golgi polarity. Related to Figure 6.

(A-C) Confocal micrographs of L3 salivary gland cells labelled for the cis-Golgi marker GM130 (magenta) and the trans-Golgi marker GCC88 (green). Glands prepared from wild type (WT) or the indicated intra-

Golgi single mutants. The insets show a higher magnification of the Golgi highlighted in the image. The dotted line illustrates a typical line profile used for quantification, with the graphs showing the normalised means of 10 line profiles of GM130 and GCC88 across the Golgi stack. Error bars, SD. Full data in Data S2.

(D-F) Confocal micrographs of L3 salivary gland cells labelled for the medial-Golgi markers Golgin-84 (green)and the trans-Golgi marker Syntaxin16 (magenta). Glands prepared from wild-type (WT), zygotic or zygotic/maternal (germ-line clone, GLC) *TMF,GMAP* double mutants. The germ-line clone ensures that there is no possible maternal contribution of the golgins to the developing progeny, and yet the Golgi is still polarised. The insets show a higher magnification of the Golgi highlighted in the image. The dotted line illustrates a typical line profile used for quantification, with the graphs showing the normalised means of 15-45 line profiles of the marker proteins across the Golgi stack. Error bars, SD. Scale bars: 2 μm. Full data in Data S2.

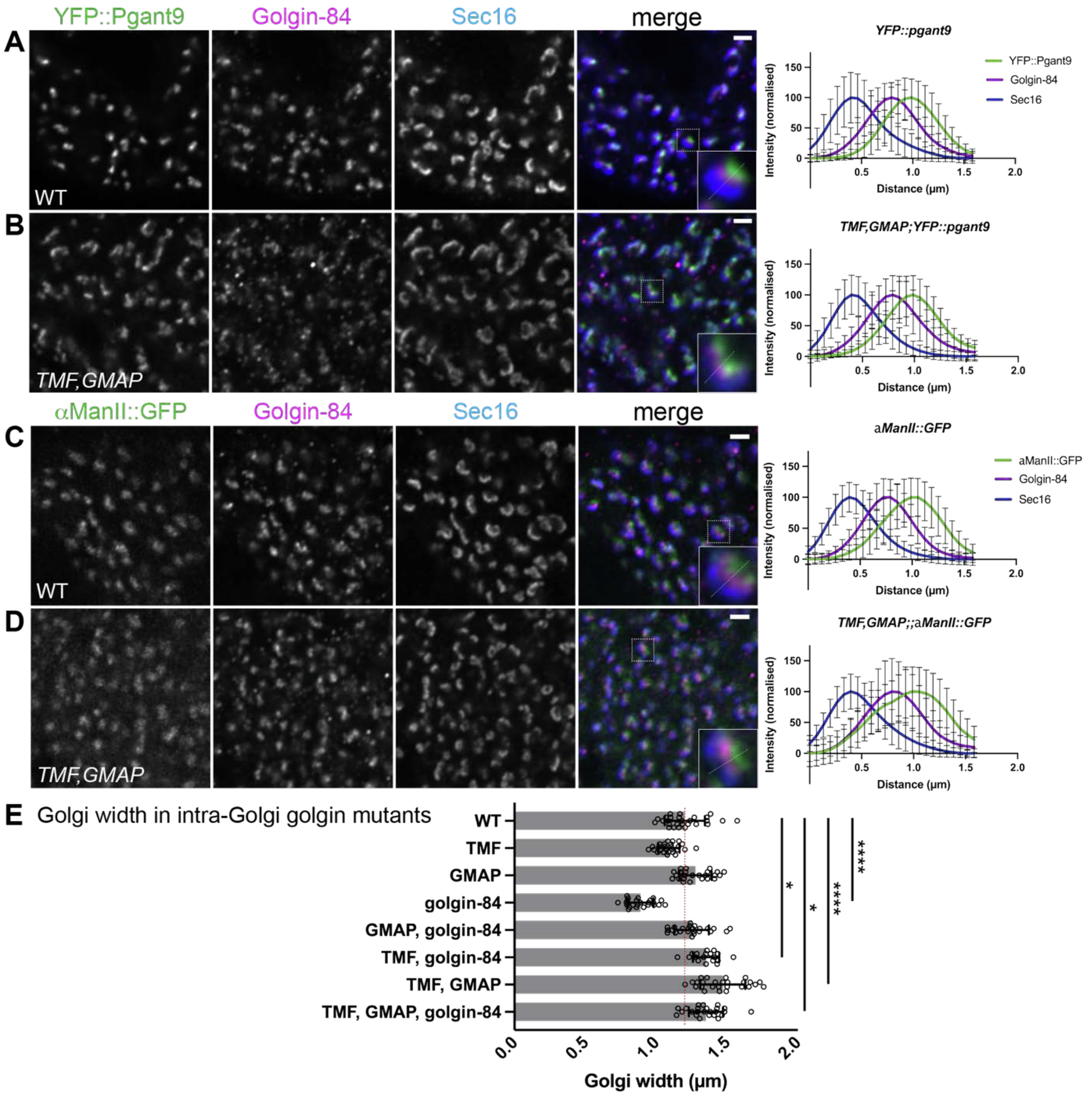

Figure S5. Characterisation of intra-Golgi golgin mutants. Related to Figure 6.

(A-D) Confocal micrographs of L3 salivary gland cells labelled for the medial-Golgi markers YFP::Pgant9 (green, A,B) or αManII::GFP (green, C,D) and Golgin-84 (magenta), and the ER exit site marker Sec16 (blue). Glands prepared from *YFP::pgant9* or *αManII::GFP* controls or *TMF,GMAP* double mutants expressing either *YFP::pgant9* or *αManII::GFP*. The insets show a higher magnification of the Golgi highlighted in the image. The dotted line illustrates a typical line profile used for quantification, with the graphs showing the normalised means of 15-45 line profiles of the marker proteins across the Golgi stack. Error bars, SD. Scale bars: 2 µm. Full Data in Data S2.

(E) Quantification of mean Golgi width in intra-Golgi golgin single, double and triple mutants using the GM130 signal for measurements (error bars are SD). 5-7 images from 3-4 larvae were analysed per genotype, and >100 Golgi measured per image. The experiment was repeated four times (three for *TMF;;*

golgin-84 double mutants). The red dotted line shows the wild type mean for comparison. Statistical significance determined by Kruskal-Wallis using Dunn's multiple comparisons, with significant difference to wild-type indicated: * = $p < 0.05$, **** = $p < 0.0001$. Full data in Data S3.

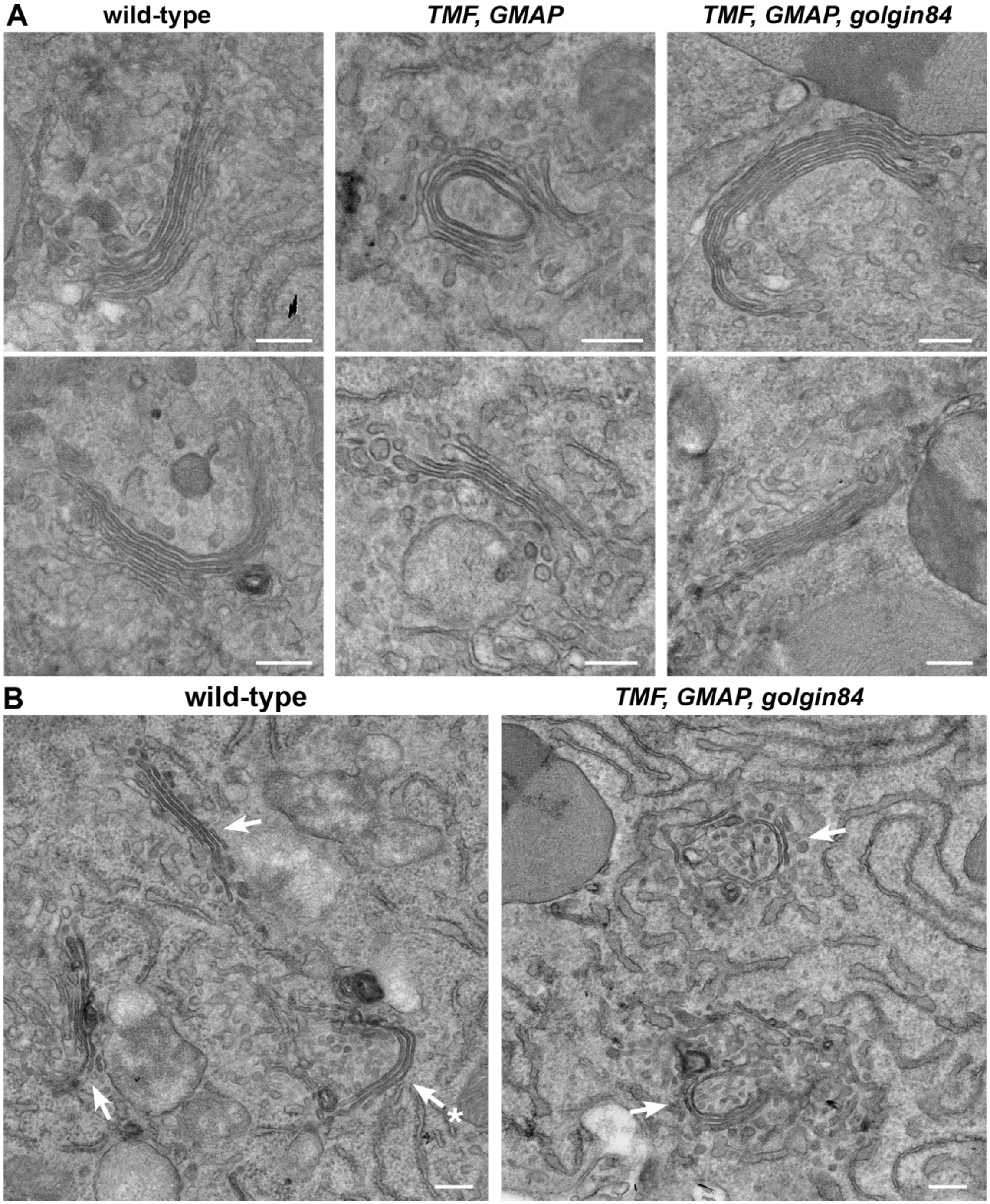

Figure S6. Golgi stacks are still formed in the absence of the intra-Golgi golgins. Related to Figure 6.

(A) Golgi stacks from electron micrographs of thin sections of L3 salivary glands from wild type or the indicated mutants. Organised stacks are still present even in the absence of all three intra-Golgi golgins. (B) Electron micrographs as in (A), illustrating the pleiomorphic nature of Golgi stacks (white arrows) in *Drosophila* tissues. The two Golgi stacks in the triple golgin mutant are associated with a large

accumulation of vesicles, but this can also be seen for some stacks in the wild type (asterisk) and so determining its significance will require further investigation by extensive morphological analysis. Scale bars 200 nm.

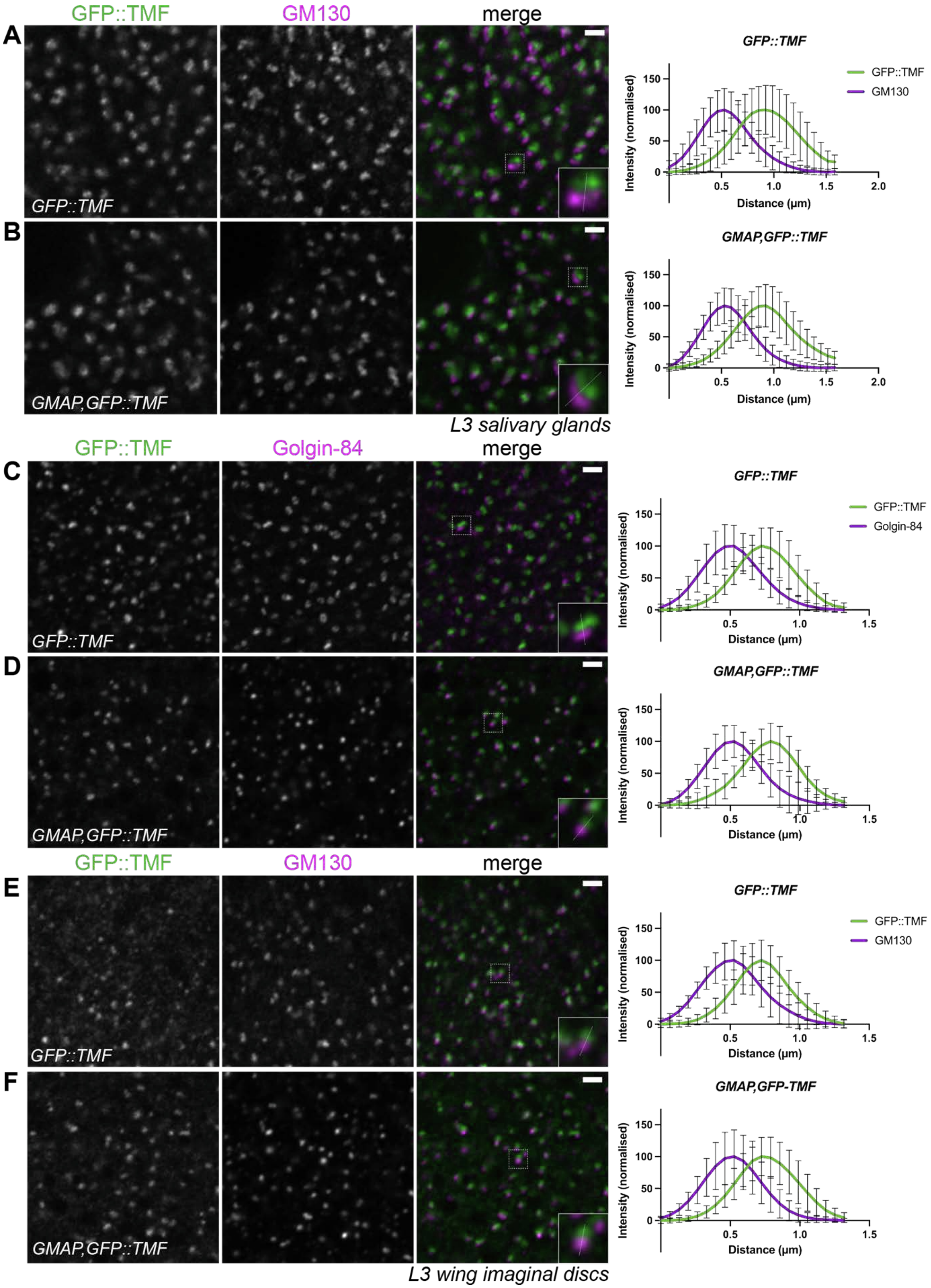

Figure S7. Loss of GMAP does not affect the localisation of GFP::TMF across the Golgi stack in L3 salivary glands or wing discs. Related to Figure 7.

(A-B) Confocal micrographs of L3 salivary gland cells labelled for GFP::TMF (green) and the cis-Golgi marker GM130 (magenta). Glands prepared from *GFP::TMF* control or *GMAP,GFP::TMF* mutants. The insets show a higher magnification of the Golgi highlighted in the image. The dotted line illustrates a typical line profile used for quantification, with the graphs showing the normalised means of 15-30 line profiles of the marker proteins across the Golgi stack. Error bars, SD. Scale bars: 2 µm. Full data in Data S2.

(C-F) Confocal micrographs of L3 wing disc cells labelled for GFP::TMF (green) and the medial-Golgi marker Golgin-84 (magenta, C-D) or the cis-Golgi marker GM130 (magenta, E-F). Glands prepared from *GFP::TMF* control or *GMAP,GFP::TMF* mutants. The insets show a higher magnification of the Golgi highlighted in the image. The dotted line illustrates a typical line profile used for quantification, with the graphs showing the normalised means of 15 line profiles of the marker proteins across the Golgi stack. Error bars, SD. Scale bars: 2 µm. Full data in Data S2.
