# Supplementary material for: In vivo characterization of Drosophila golgins reveals redundancy and plasticity of vesicle capture at the Golgi apparatus
Source: Curr Biol. 2022 Nov 7;32(21):4549–4564.e6. doi: 10.1016/j.cub.2022.08.054 (PMC9849145; doi:10.1016/j.cub.2022.08.054)
Supplement: Document S2. Article plus supplemental information [file mmc5.pdf]

Current Biology

Article

# *In vivo* characterization of *Drosophila* golgins reveals redundancy and plasticity of vesicle capture at the Golgi apparatus

## Graphical abstract

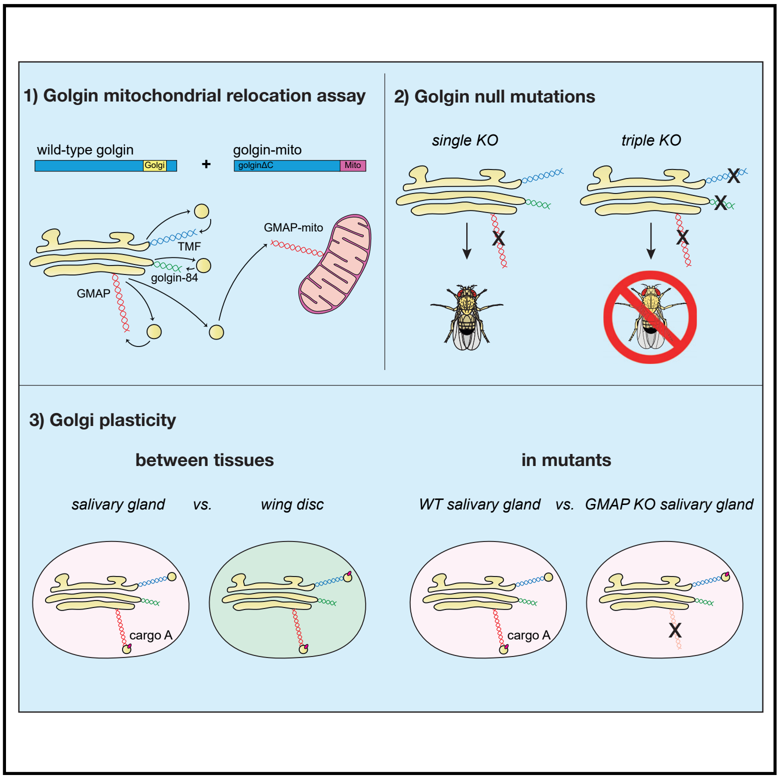


## Highlights

- Three *Drosophila* golgins can capture intra-Golgi transport vesicles in tissues
- The content of the vesicles captured varies between golgins and between tissues
- Null mutations in the golgins have mild phenotypes but become severe when combined
- A change in vesicle content in a golgin mutant reveals Golgi plasticity

## Authors

Sung Yun Park, Nadine Muschalik, Jessica Chadwick, Sean Munro

## Correspondence

sean@mrc-lmb.cam.ac.uk

## In brief

Golgin coiled-coil proteins capture the vesicles that recycle Golgi residents. Park et al. relocate three *Drosophila* golgins to mitochondria to show that the content of intra-Golgi vesicles varies between tissues and in a golgin mutant. Such plasticity of Golgi organization could explain the synergistic effects of combining golgin null mutants.

Park et al., 2022, Current Biology *32*, 4549–4564
November 7, 2022 © 2022 The Author(s). Published by Elsevier Inc.
https://doi.org/10.1016/j.cub.2022.08.054

Current Biology

CellPress
OPEN ACCESS

Article

# *In vivo* characterization of *Drosophila* golgins reveals redundancy and plasticity of vesicle capture at the Golgi apparatus

Sung Yun Park,[1,2] Nadine Muschalik,[1,2] Jessica Chadwick,[1] and Sean Munro[1,3,*]
[1]MRC Laboratory of Molecular Biology, Francis Crick Avenue, Cambridge CB2 0QH, UK
[2]These authors contributed equally
[3]Lead contact
*Correspondence: sean@mrc-lmb.cam.ac.uk
https://doi.org/10.1016/j.cub.2022.08.054

## SUMMARY

The Golgi is the central sorting station in the secretory pathway and thus the destination of transport vesicles arriving from the endoplasmic reticulum and endosomes and from within the Golgi itself. Cell viability, therefore, requires that the Golgi accurately receives multiple classes of vesicle. One set of proteins proposed to direct vesicle arrival at the Golgi are the golgins, long coiled-coil proteins localized to specific parts of the Golgi stack. In mammalian cells, three of the golgins, TMF, golgin-84, and GMAP-210, can capture intra-Golgi transport vesicles when placed in an ectopic location. However, the individual golgins are not required for cell viability, and mouse knockout mutants only have defects in specific tissues. To further illuminate this system, we examine the *Drosophila* orthologs of these three intra-Golgi golgins. We show that ectopic forms can capture intra-Golgi transport vesicles, but strikingly, the cargo present in the vesicles captured by each golgin varies between tissues. Loss-of-function mutants show that the golgins are individually dispensable, although the loss of TMF recapitulates the male fertility defects observed in mice. However, the deletion of multiple golgins results in defects in glycosylation and loss of viability. Examining the vesicles captured by a particular golgin when another golgin is missing reveals that the vesicle content in one tissue changes to resemble that of a different tissue. This reveals a plasticity in Golgi organization between tissues, providing an explanation for why the Golgi is sufficiently robust to tolerate the loss of many of the individual components of its membrane traffic machinery.

## INTRODUCTION

The Golgi apparatus is the central sorting station in the secretory pathway and the connection between the exocytic and endocytic systems. It is also the site of extensive posttranslational modification of proteins and lipids, particularly the formation and elaboration of glycan structures attached to glycoproteins and glycolipids.[1–3] The Golgi comprises a stack of flattened cisternae, starting with the *cis*-Golgi where vesicles arrive from the endoplasmic reticulum (ER) and ending with the *trans*-Golgi network (TGN) from which carriers depart to the cell surface or compartments of the endocytic system.[4,5] The Golgi resident membrane proteins that direct membrane traffic or posttranslational modifications are distributed across specific parts of the stack. These resident proteins remain within the stack despite the flow of cargo through the Golgi to elsewhere in the cell.[6–8] It is now widely accepted that these residents are retained by recycling in vesicles as the cisternae progress and mature from the *cis* to *trans* side.[9–11] However, how this recycling works remains unclear, especially as only one type of vesicle coat appears to be involved, the coat protein complex I (COPI) coat formed of coatomer subunits.

Retrograde traffic within the Golgi stack requires not only machinery to make vesicles but also a mechanism to ensure that the recycling vesicles are captured by specific earlier cisternae to which they then fuse.[12,13] One class of proteins that has been proposed to act in this process are the golgins—a family of long coiled-coil proteins that localize to specific parts of the stack and are well conserved in evolution.[14–16] Evidence that the golgins can act as specific vesicle tethers has come from experiments in mammalian tissue culture cells, where it was found that when the golgins are relocated to an ectopic location, the mitochondria, each ectopic golgin can capture a specific class of Golgi-bound vesicles.[17–19] Of the ten mammalian golgins, three are able to capture vesicles that contain Golgi resident proteins. These intra-Golgi golgins are Golgi microtubule-associated protein 210 (GMAP-210), golgin-84, and TATA element modulatory factor (TMF).[20–22] Although they all capture vesicles containing Golgi resident membrane proteins, there are differences in the specific Golgi proteins in the captured vesicles. TMF captures proteins from later compartments in the stack than the other two golgins, consistent with TMF being located toward the *trans* side, and golgin-84 and GMAP-210 being located on the *cis* side.[23–25] For all three golgins, a short and well-conserved region at the N terminus is sufficient for vesicle capture.[26] The conserved features of these regions vary between the three proteins, but all start with a similar motif (M-S-W-F/L),


This is an open access article under the CC BY license (http://creativecommons.org/licenses/by/4.0/).

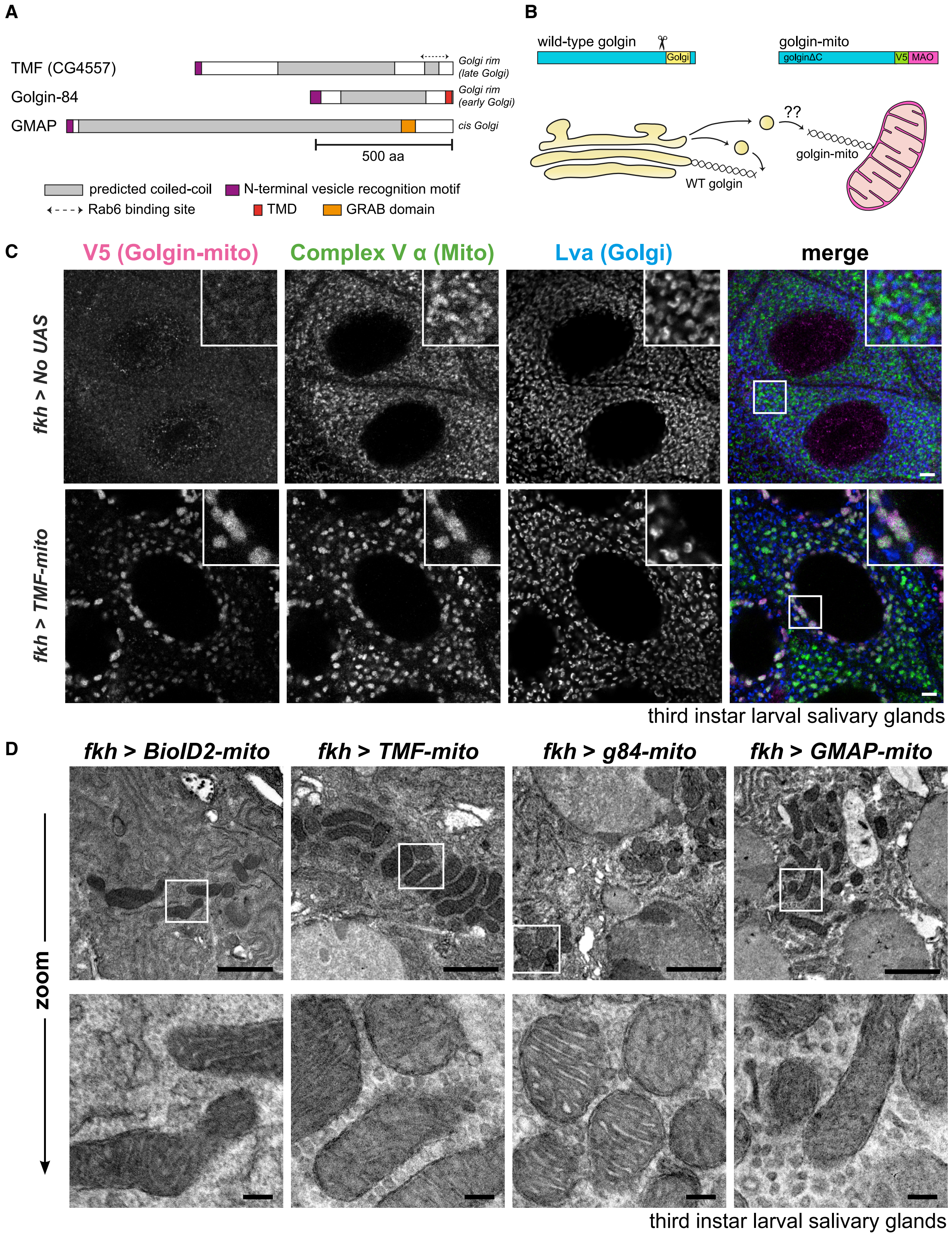


**Figure 1. Tissue-specific golgin mitochondrial relocation strategy in *Drosophila melanogaster***

(A) Schematic of the *Drosophila* intra-Golgi golgins. Coiled-coil regions were predicted using a 28-residue window, other domains are as indicated. TMF is located more toward the late Golgi than are GMAP and Golgin-84.

(legend continued on next page)

CellPress
OPEN ACCESS

suggesting that they act by recognizing vesicles by distinct but related mechanisms.

Although there is good evidence that these golgins are sufficient for vesicle capture, it remains unclear how important they are for cell function.[27] Application of gene knockdown or knockout in cell culture indicates that none are essential for cell viability, which is perhaps surprising given that all three are conserved from mammals to plants and fungi. In addition, mice lacking golgin-84 are viable and fertile, and those lacking TMF reach adulthood, although the males are sterile, with defects in acrosome formation during spermatogenesis.[28,29] Loss of GMAP-210 causes embryonic lethality with aberrant bone formation arising from defects in the deposition of extracellular matrix by osteoblasts and chondrocytes.[30,31] However, interpreting this phenotype is complicated by GMAP-210 having been found to also capture ER to Golgi carriers, with defective bone formation being a feature of mutants in several components of ER to Golgi trafficking.[17,32–34] The lack of more severe phenotypes for mutations in these well-conserved golgins may reflect some degree of functional redundancy, and indeed there is considerable overlap in the sets of Golgi residents present in the vesicles that they capture.[17,26] However, there have been no previous reports on the effects of deleting combinations of these three golgins in a whole animal.

We have thus used the *Drosophila* system to address the role of these three golgins in the diverse tissues of metazoans. Almost all known components of mammalian membrane traffic are well conserved in *Drosophila*, including all three of these golgins, and genetic and cell biological studies in flies have proven valuable for illuminating the function of other membrane traffic components.[35–40] We show that the mitochondrial relocation assay that we used in tissue culture cells to demonstrate that the golgins act as vesicle tethers, can be applied to *Drosophila* tissues to interrogate the content of the vesicles captured by a particular golgin, and we test if the content of the vesicles captured by a specific golgin is invariant between tissues. We then generate loss-of-function alleles for each of the golgins and examine the effects of combining these mutations to determine the degree of functional redundancy between the intra-Golgi golgins.

## RESULTS

### Validation of a tissue-specific golgin mitochondrial relocation system in *Drosophila*

The overall architecture of the three mammalian golgins that capture intra-Golgi transport vesicles is well conserved in their *Drosophila* orthologs, with each golgin possessing a long coiled-coil region sandwiched by a highly conserved C-terminal Golgi localization domain and an N-terminal vesicle tethering region (Figure 1A). The N-terminal vesicle tethering regions, including the intra-Golgi golgin signature motif (M-S-W-F/L), are conserved between all three *Drosophila* golgins and their mammalian counterparts.[26] Intriguingly, the relative lengths of the coiled-coil regions of golgins are well preserved across metazoan species despite the sequence being poorly conserved.[41] Like their mammalian counterparts, Golgin-84 and GMAP are localized at the *cis* side of the Golgi.[42,43] TMF has not been previously characterized in *Drosophila*, and so we inserted GFP into the N-terminal region of the endogenous gene with CRISPR-Cas9-mediated homology-directed repair. The resulting GFP-TMF is localized between the *cis* marker GM130 and the *trans*-Golgi markers Golgin-245 and syntaxin 16 (Figure S1A). Thus, like its mammalian ortholog, TMF is localized further toward the *trans* side than either GMAP or Golgin-84.

We next determined whether we could interrogate the vesicle capture activity of the *Drosophila* golgins in specific tissues by applying the mitochondrial relocation assay that we had previously applied successfully in mammalian tissue culture cells.[17] Thus, we used the GAL4-UAS system to express in specific tissues a form of each *Drosophila* golgin in which the C-terminal Golgi localization domain was replaced with an epitope tag and the transmembrane domain (TMD) of the human mitochondrial protein, monoamine oxidase (MAO) (Figure 1B). The golgin-mito chimeras were initially expressed in the salivary glands of third instar larvae (L3), using the *forkhead-GAL4* (fkh-GAL4) driver. This tissue was chosen due to its highly secretory nature and stereotyped cuboidal epithelial monolayer. The golgin-mito chimeras, along with a similar fusion to an irrelevant control protein with no conceivable role in membrane traffic (BioID2), all showed efficient relocation to the mitochondria and loss of Golgi targeting (Figures 1C and S1B).

### Mitochondrial forms of TMF, Golgin-84, and GMAP are *bona fide* vesicle tethers in *Drosophila*

The ectopic golgins were tested for vesicle tethering activity by performing transmission electron microscopy (TEM) on salivary glands from third instar larvae. In contrast to BioID2-mito, the TMF, Golgin-84, and GMAP-mito chimeras each caused a striking accumulation of vesicular profiles around mitochondria (Figure 1D), similarly to what was observed in mammalian cells.[17] Mitochondrial clustering was observed in both EM and immunofluorescence images (Figures 1C, 1D, and S1B). Such clustering was also observed in golgin-mito expressing mammalian cells and presumably reflects vesicle capture causing adjacent mitochondria to zip together. Taken together, these data confirm the

(B) Strategy for tissue-specific relocation of golgins to mitochondria. A golgin ("golgin-mito") is relocated to the mitochondria by replacement of its C-terminal Golgi-targeting domain (yellow box) with the TMD of monoamine oxidase (MAO).

(C) Confocal micrographs of the proximal cells of L3 salivary glands labeled for the V5 epitope tag in the golgin-mito (magenta), mitochondrial complex V $\alpha$ (green), and Golgi marker Lava lamp (Lva; blue). Insets show zooms of the boxed regions in the merge. Proximal cells were chosen as they do not contain large glue granules, and so the Golgi is more clearly visible. One golgin-mito (TMF-mito) and the negative control (no UAS) are shown. See Figure S1B for micrographs of the other golgin-mito constructs and quantification of the data.

(D) Transmission electron micrographs evidencing vesicle tethering by all intra-Golgi golgin-mito chimeras in L3 salivary glands. BioID2-mito is used as a negative control. Representative micrographs are shown from 4 sections obtained from each of 8 larvae per genotype. Bottom row: zoom-in views of boxed regions in micrographs above. (C and D) Scale bars: 5 μm in (C), 1 μm in (D, top), and 100 nm in (D, bottom). See also Figure S1.

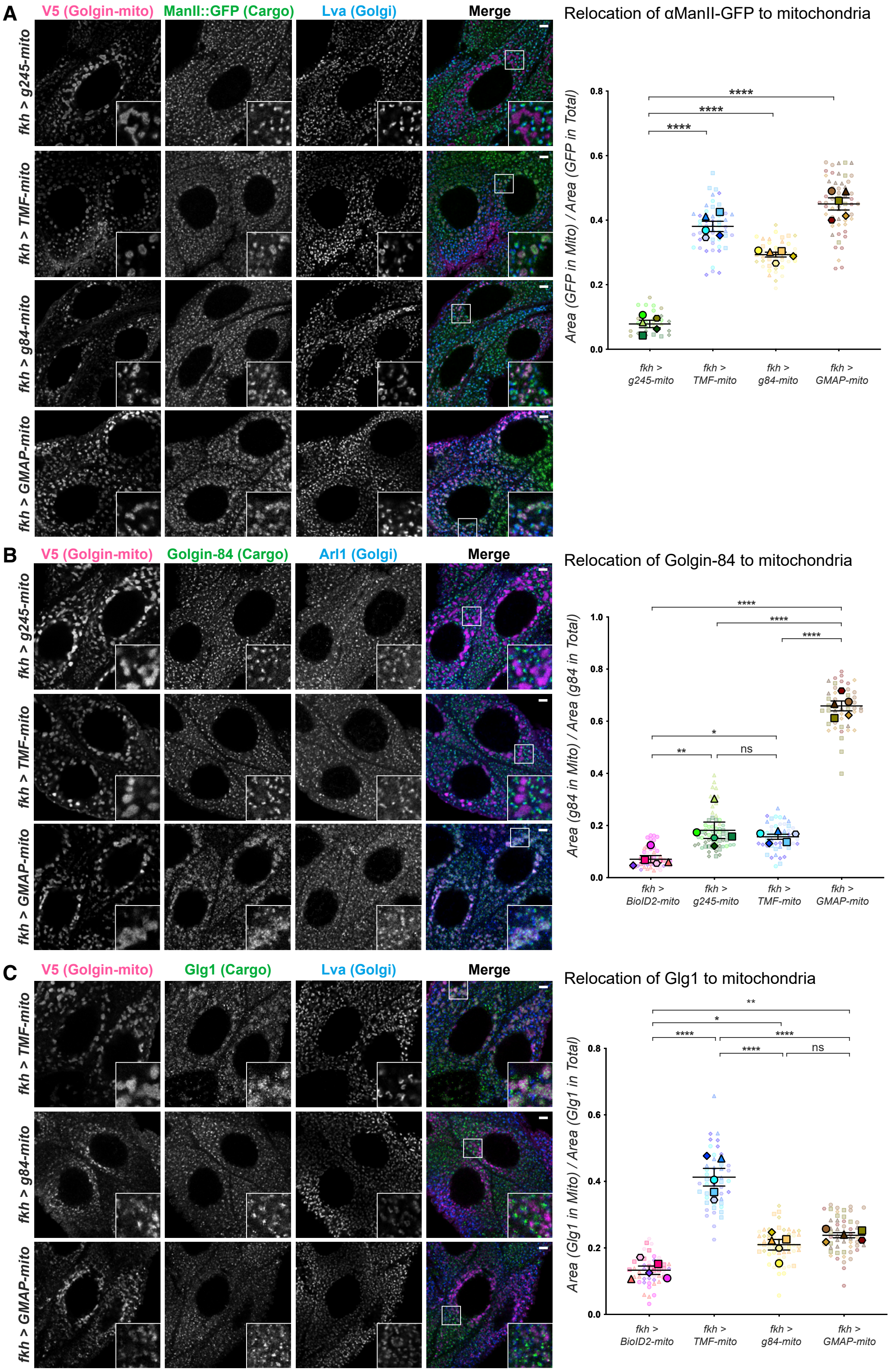


**Figure 2. *Drosophila* intra-Golgi golgin-mitos capture distinct subclasses of intra-Golgi vesicles in larval salivary glands**

(A) Left: confocal micrographs of L3 salivary glands expressing the indicated V5-tagged golgin-mito constructs and labeled for V5 (magenta), endogenous αManII::GFP (green), and Golgi marker Lva (blue). The endosome-to-Golgi tether Golgin-245-mito is used as an additional negative control to demonstrate that

(legend continued on next page)

successful reconstitution of the golgin mitochondrial relocation system in *Drosophila*.

## TMF, Golgin-84, and GMAP capture distinct subclasses of intra-Golgi vesicles

We next applied the mitochondrial relocation assay to compare the content of the vesicles captured by each of the intra-Golgi golgins. We first examined the proximal cells of L3 salivary glands, assaying for the relocation of the resident membrane proteins of the Golgi, which are the cargo of intra-Golgi transport vesicles. The Golgi in most *Drosophila* cells are present as multiple individual stacks rather than the single perinuclear ribbon found in many mammalian cell types, but otherwise they perform an identical role in membrane traffic and glycosylation and contain many of the same proteins.[35,44] Golgi residents were investigated using antibodies against endogenous proteins, such as Glg1 and Golgin-84 itself (it possesses a TMD and so must recycle within the stack), or using lines with the Golgi enzymes α-Mannosidase II a (αMan-II) and Pgant9 tagged in the genome with fluorescent proteins.

All three of the golgins were able to relocate the major Golgi glycosylation enzyme αManII to the mitochondria (Figure 2A). The relocated αManII::GFP is likely to be in Golgi-derived carriers rather than in whole Golgi fragments, as the Golgi marker Lava lamp (Lva) was not relocated by any of the golgin-mito chimeras.[45] By contrast, mitochondrially relocated forms of the negative control BioID2, or of the *trans*-Golgi golgin Golgin-245, an endosome-to-Golgi vesicle tether, exhibited little to no αManII::GFP relocation. Quantitation of the degree of relocation of αManII::GFP to mitochondria confirmed these effects and showed that all three intra-golgins capture similar amounts of the cargo (Figure 2A). Taken together, these data indicate that *Drosophila* TMF, Golgin-84, and GMAP are all able to capture intra-Golgi vesicles, and so the role of this golgin subset is conserved between insects and mammals.

Despite shared capture of αManII, the three intra-Golgi golgins displayed clear differences in their specificities for the cargoes Golgin-84 and Glg1. In the case of Golgin-84, GMAP-mito exhibited significantly greater Golgin-84 cargo capture activity than TMF-mito (Figures 2B and S2A). Whether Golgin-84 itself captures recycling Golgin-84 could not be determined due to a lack of appropriate tools. Intriguingly, a reversed capture pattern was observed for Golgi resident protein Glg1, which was most efficiently relocated by TMF-mito among the intra-Golgi golgin subset (Figures 2C and S2B). In contrast to the Golgin-84 and Glg1 cargo capture phenotypes, intra-Golgi golgins displayed more subtle differences in YFP::Pgant9 cargo capture. All three golgins demonstrated significantly higher YFP::Pgant9 capture than the control Golgin-245-mito, yet differed in the degree of capture efficiency (Figure S2C). GMAP-mito showed the strongest YFP::Pgant9 capture efficiency, whereas TMF-mito and Golgin-84-mito had weaker efficiency (Figure S2C). Overall, the combined data indicate that the intra-Golgi golgins possess golgin-specific cargo capture functions alongside some shared activities, and hence they must differ in affinities for specific subsets of intra-Golgi vesicles.

## Golgin tethering functions are not invariant between tissues

We next tested whether the pattern of cargo captured by the golgins is cell type dependent by assaying the same cargoes in a second tissue, the L3 wing disc. In these experiments, engrailed-GAL4 was used to drive expression of the golgin-mito proteins in the posterior compartment of the disc. As in the salivary gland, the abundant Golgi enzyme αManII was captured efficiently by both GMAP-mito and TMF-mito, whereas the Golgi marker Lva was not perturbed (Figure 3A). By contrast, expression of Golgin-84-mito in the wing discs gave little, if any, relocation of αManII, despite showing efficient relocation of this protein in salivary glands. Golgin-84 is expressed in wing discs (Figure S2D), and examining the relocation of Golgin-84 by the other two golgins revealed a second difference to the findings from salivary glands. In salivary glands GMAP, but not TMF, gave efficient relocation of Golgin-84, but in contrast, in wing discs Golgin-84 was relocated efficiently by both constructs (Figure 3B). This indicates that the carriers captured by TMF contain Golgin-84 in wing discs but not in salivary glands.

These differences between the salivary gland and the wing disc were recapitulated with the other Golgi markers. In salivary glands, Glg1 was relocalized most efficiently by TMF, with less relocation by Golgin-84 and GMAP, and in wing discs, there was again efficient capture only by TMF and no capture at all by Golgin-84 (Figures 3C and S2E). Finally, Pgant9 showed both an increase in relative capture by TMF, compared with GMAP, and a loss of capture by Golgin-84 in wing discs versus salivary glands (Figure S2F). In summary, there are major differences in the pattern of cargo capture seen in wing discs and salivary glands. Firstly, in wing discs, Golgin-84 does not capture cargoes recruited by this golgin in salivary glands, while little to no changes were observed for GMAP-mito between the two tissues. Secondly, TMF captures some cargo efficiently in wing discs but not in salivary glands.

capture of Golgi-derived vesicles is specific to intra-Golgi golgins. Insets show zooms of the boxed regions in the merge. (A) Right: quantification of αManII::GFP relocation using ratios between area of cargo in mitochondria and total area of mitochondria.

(B) As in (A), except labeled for endogenous Golgin-84 (green) and *trans*-Golgi marker Arl1 (blue). Quantifications were obtained by calculating the ratio between area of cargo in mitochondria to total area of cargo. GMAP-mito captures Golgin-84 more efficiently than TMF-mito. Golgin-84-mito was not tested as it is labeled by the Golgin-84 antibody.

(C) As in (A), except labeled for endogenous Glg1 (green) and the Golgi marker Lva (blue). Quantifications were obtained by calculating the ratio between area of cargo in mitochondria to total area of cargo, demonstrating specificity of TMF-mito for Glg1-containing vesicles among the intra-Golgi golgin subset (left). Scale bars: 5 μm. For quantifications in (A)–(C), five larvae were examined with 2–17 cells segmented in each and quantified per cell. The individual cell ratios are shown with small, partially transparent, symbols, and the mean for each of the five larvae shown as large opaque points. Points are shaped and colored according to larval replicate, with the coding scheme of cell-by-cell points matching that of their corresponding larval points. The mean larval ratio (line) and SEM (bars) are plotted for each genotype. Data analyzed by one-way nested ANOVA using Tukey's multiple comparisons, ns, not significant, *$p \leq 0.05$, **$p \leq 0.01$, and ****$p \leq 0.0001$. See also Figure S2 and Data S1.

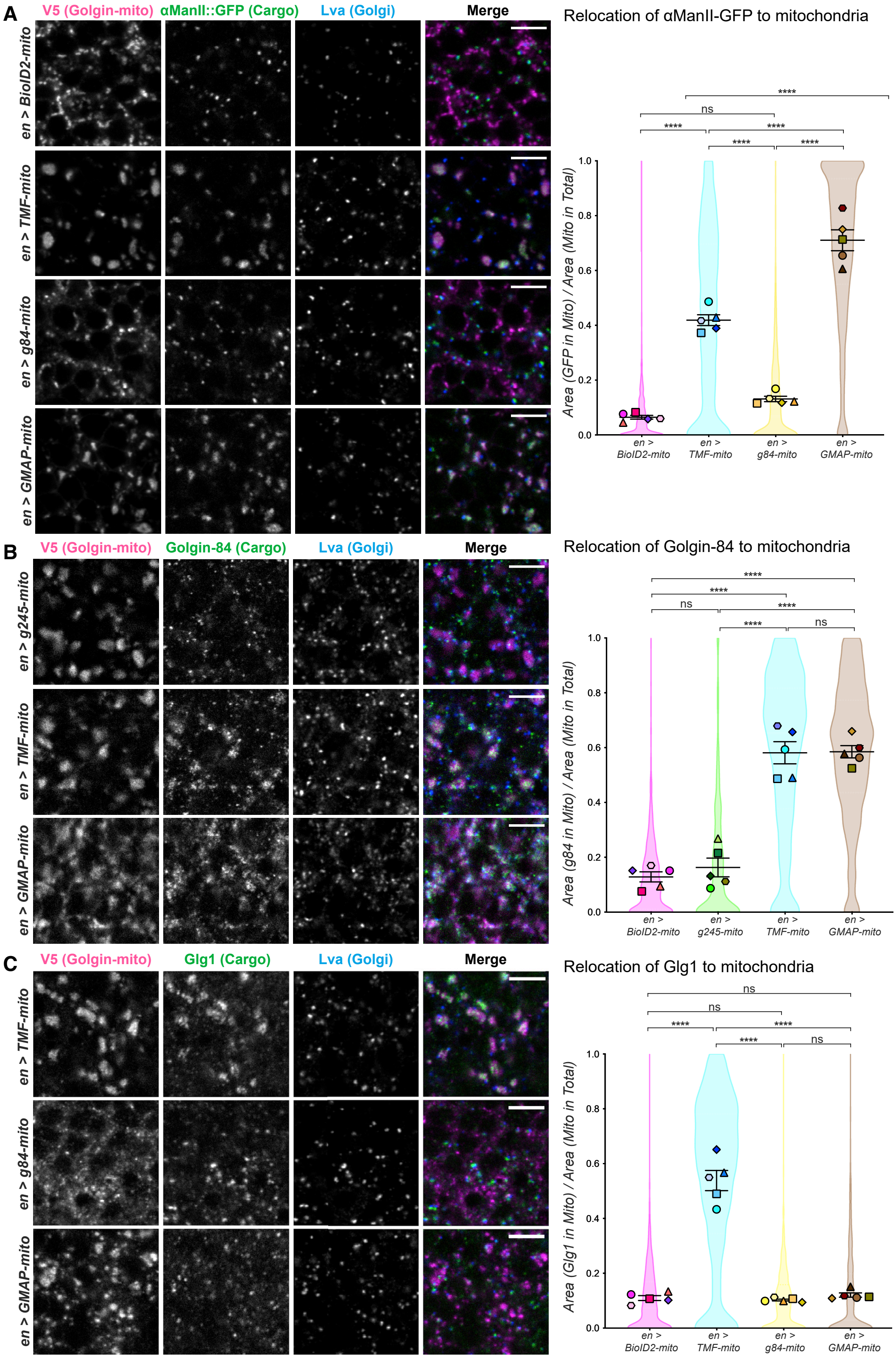


(legend on next page)

## Generation of *TMF*, *GMAP*, and *golgin-84* mutant flies

The above results indicate that the three intra-Golgi golgins share the ability to capture intra-Golgi transport vesicles, even though the activity of each golgin and the content of the vesicles that they capture can vary considerably between tissues. However, although these golgins can clearly capture vesicles, the biological importance of this activity remains unclear. To investigate the importance of TMF, GMAP, and Golgin-84 in flies, and to test for redundancy between these golgins, we generated mutants using the CRISPR-Cas9 system (Figure 4).

*GMAP* is predicted to encode four isoforms that differ in their untranslated regions and N termini. To target all isoforms, we generated a 4-bp deletion at the start of exon 3 to create *GMAP_2.2.5* (Figure 4A). The full-length GMAP is absent in *GMAP_2.2.5*, although a shorter form appears at low levels (Figure 4D), as is often seen with CRISPR-Cas9-generated indels.[46] This form will have lost the N-terminal vesicle tethering region and is substantially less abundant than the wild type (WT); thus, if *GMAP_2.2.5* is not a null, it is a very strong hypomorph. For both *TMF* and *golgin-84*, we used pairs of guide RNAs to delete all or part of the coding region (Figures 4B and 4C). The allele *TMF_Δ19* carries a precise deletion of 3,239 bp that removes the entire coding region and is thus an amorphic allele (Figures 4B and S3A). In the *golgin-84_Δ13.3* allele, the first 178 residues are deleted, including its predicted N-terminal vesicle capturing motif, and no residual band was detected by immunoblotting, indicating that *golgin-84_Δ13.3* is also a null allele (Figures 4C and 4E).

## *TMF*, *GMAP* and *golgin-84* mutant flies are viable, but the loss of TMF affects male fertility

Female flies with homozygous mutations in the three individual golgins were all viable and fertile, as were males lacking GMAP or Golgin-84. However, *TMF* mutant males exhibited partial male sterility (Figure 5A). Interestingly, mice lacking TMF are also viable but the males are infertile as their spermatids lack the acrosome, a specialized Golgi-derived compartment that is essential for fertilization, and in addition, the cytoplasm is not properly removed from the spermatids leading to misshapen and immotile spermatozoa.[29] Acrosome formation in mammals appears highly dependent on aspects of Golgi function, being also dependent on other Golgi proteins such as golgin-160 and GM130.[47,48] We thus examined two markers of the *Drosophila* acrosome, Snky-GFP and GFP-LAMP (lysosome-associated membrane glycoprotein). The loss of TMF did not prevent the normal accumulation of these markers next to the sperm nucleus where the acrosome is located, but in developing spermatids, GFP-LAMP showed an increased accumulation in structures throughout the cytoplasm, indicating a partial defect in sorting of membrane proteins to the forming acrosome (Figures 5B–5G). In addition to the sterility phenotype, we also noticed abnormalities in testis shape in a fraction of the mutant flies (Figure 5H–5K'; 4/27, 14.8%). The affected testes failed to develop normally and retained a more pupal-like shape in *TMF* mutant adults, suggesting a role for TMF in testis morphogenesis, which may also account for the reduced male fertility. Taken together, the data show that most cell types appear to be able to function normally in the absence of any one of the intra-Golgi golgins, although TMF has a particularly important role in both spermatogenesis and testis development.

## Combining golgin mutants results in synergistic defects

Mutations in each of the three golgins result in mild phenotypes despite the proteins being well conserved in evolution and expressed in most, if not all, tissues. However, there is considerable overlap in the set of cargo present in the vesicles that they capture, and so to test for redundancy, we generated all possible double mutants and the triple mutant. Mutants lacking both GMAP and Golgin-84 are viable and fertile with no discernible morphological phenotypes. Similarly, zygotic *TMF,golgin-84* double mutants are also viable with no obvious phenotype apart from the male infertility that was also seen in *TMF* mutants. By contrast, zygotic *TMF,GMAP* mutants are lethal, with all of the flies dying during, or shortly after, eclosion (Figures 5L and 5M). Finally, in the triple mutant lacking TMF, GMAP, and Golgin-84, the lethality shifts to earlier stages with most animals dying as pupae and others that do eclose dying shortly afterward (Figure 5N).

Examining the testes in the pupae formed by the mutants lacking both TMF and GMAP revealed severe and fully penetrant defects in morphology that exceeded those seen with TMF alone, with no additional effect being seen upon removal of Golgin-84 (Figures 5O and 5P). Testis development relies on a sheath of myofibers that form at L3 to shape the initial small sphere of cells into the distinctive spiral of mature tissue (Figure 5I).[49,50] Staining this muscle sheath with phalloidin revealed no defects in the *GMAP* and *golgin-84* single and double mutants, showing only occasional irregularities in the *TMF* single and *TMF;;golgin-84* double mutants (Figures 5Q, 5R, S3B–S3D, S3G, and S3H). By contrast, combining the *TMF* and *GMAP* mutations resulted in severe morphological defects with defective elongation and either missing or perforated muscle sheaths (Figures 5S, 5T, and S3E). No additive effect of Golgin-84 in testis development, and thus muscle sheath formation, was seen in the triple mutant

**Figure 3. Intra-Golgi golgin vesicle tethering activities are altered in larval wing imaginal discs**

(A) Confocal micrographs of L3 wing imaginal discs expressing the indicated V5-tagged golgin-mito constructs and labeled for V5 (magenta), endogenous αManII::GFP (green), and Golgi marker Lva (blue). Insets show enlargements of the boxed regions in the merge, and quantification of experiment is shown on the right.

(B) As in (A), except labeled for V5 (magenta), endogenous Golgin-84 (green), and the Golgi marker Lva (blue). TMF-mito efficiently captures Golgin-84 cargo in contrast to what was observed in larval salivary glands.

(C) As in (A), except labeled for V5 (magenta), endogenous Glg1 (green), and Golgi marker Lva (blue). Scale bars: 5 μm. Quantifications in (A)–(C) were obtained by calculating the ratio between area of cargo in mitochondria to total area of mitochondria. Cell-by-cell ratios from five discs each from a different larva were pooled and are shown as truncated violin plots. Ratios for discs (averaged cell-by-cell ratios) are plotted as large opaque points. The cells in discs were segmented and quantified with 68–495 cells depending on the disc. The mean larval ratio (line) and SEM (bars) are plotted for each genotype. Data analyzed by one-way nested ANOVA using Tukey's multiple comparisons, ns, not significant, *$p \leq 0.05$, **$p \leq 0.01$, ***$p \leq 0.001$, and ****$p \leq 0.0001$. Full data in Data S1 and see also Figure S2.

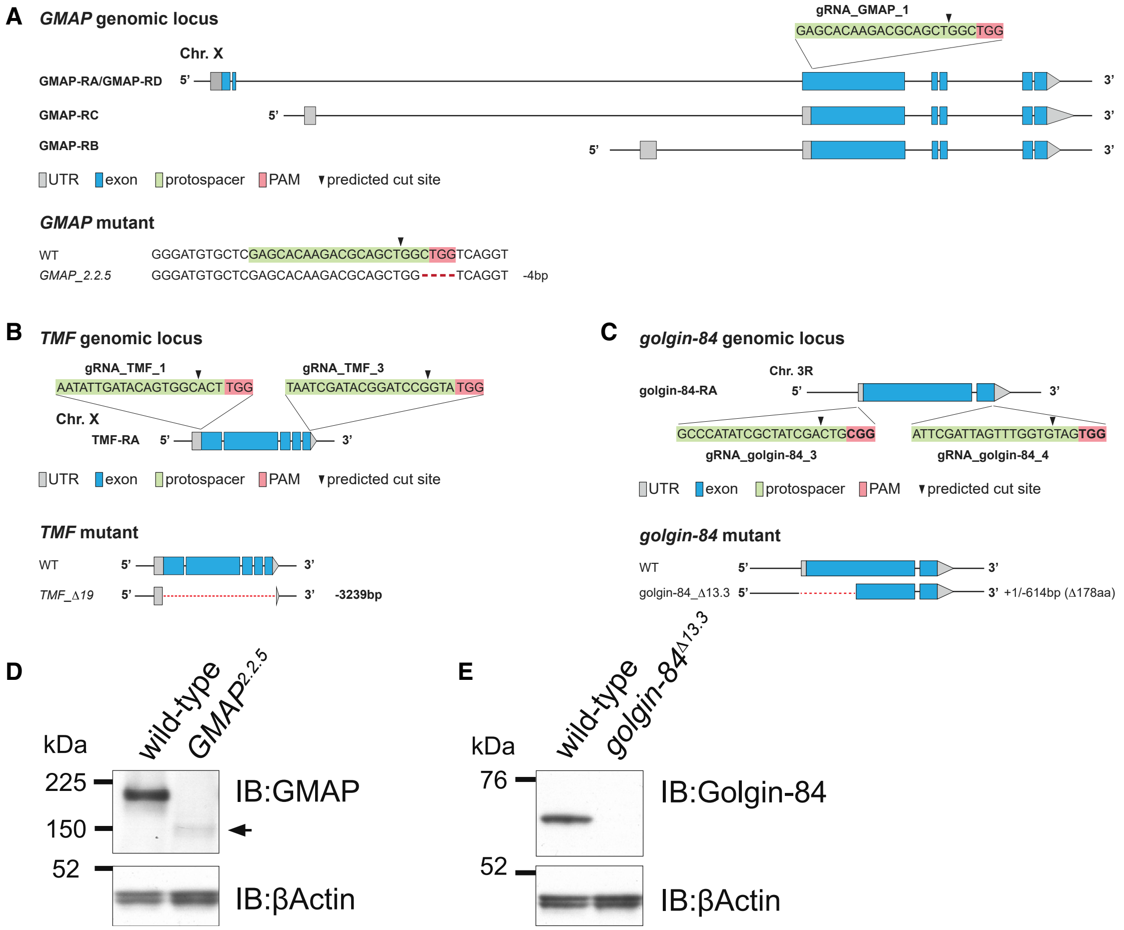


**Figure 4. Generation of mutants in the intra-Golgi golgins using CRISPR-Cas9**

(A) Schematic of the *Drosophila GMAP* genomic locus. A gRNA targets the 5′ end of exon 3 to mutate all potential isoforms. The resulting allele *GMAP_2.2.5* carries a 4-bp deletion.

(B) Schematic of the *Drosophila TMF* genomic locus. A pair of gRNAs was designed to delete the coding region with small parts of both UTRs remaining. Allele *TMF_Δ19* carries a precise deletion of 3,239 bp.

(C) Schematic of *Drosophila golgin-84*. Two gRNAs were designed to delete the coding region, with the resulting allele *golgin-84_ Δ13.3* having a +1 bp/−614 bp indel resulting in a protein lacking the N-terminal 178 residues, including the vesicle tethering motif.

(D) Immunoblot to confirm that *GMAP_2.2.5* is a strong hypomorph. The arrow highlights a weak band of ~150 kDa that appears in the mutant. The GMAP antibody was raised against residues 778–1,057.[42]

(E) Immunoblot to show the loss of Golgin-84 in the *golgin-84_ Δ13.3* mutant, confirming that it is a null allele. The antibody was raised against residues 1–300.[42] See also Figure S3.

(Figures 5T and S3F). This suggests that TMF and GMAP share a function that is critical for development of this muscle sheath. Taken together, these genetic data demonstrate that combinations of mutants in the intra-Golgi golgins give much stronger phenotypes than the single mutants, particularly those of *GMAP* and *TMF*, and that the intra-Golgi golgins are collectively essential for viability.

## Effects of golgin mutants on Golgi structure and function

We next examined the effects of the golgin mutants at a cellular level. As stated above, in most cell types the *Drosophila* Golgi exists as multiple stacks located throughout the cytoplasm, and markers at the *cis* and *trans* ends can be readily resolved by light microscopy.[35] GM130 (*cis*) and GCC88 (*trans*) remained well resolved in larval salivary glands from both the single and combined mutants, indicating that the polarized organization of the stacks is not grossly perturbed by the loss of golgins (Figures 6A–6E and S4A–S4C). Similar results were obtained with other Golgi markers, including integral membrane proteins (Figures S4D–S4F and S5A–S5D). The width of the Golgi stacks was affected in some of the mutants with the loss of Golgin-84 causing a consistently narrower stack, and the *TMF,GMAP* double mutant having slightly wider stacks (Figure S5E). The reasons for these changes are unclear, but a decrease in Golgi size was also observed in a mutant lacking Cog7,[39] a subunit of a putative vesicle tethering complex for intra-Golgi vesicles, and perturbations in vesicle recycling seem quite likely to lead to changes in the dynamics of cisternal formation and maturation. Electron microscopy of thin sections from larval salivary glands showed that

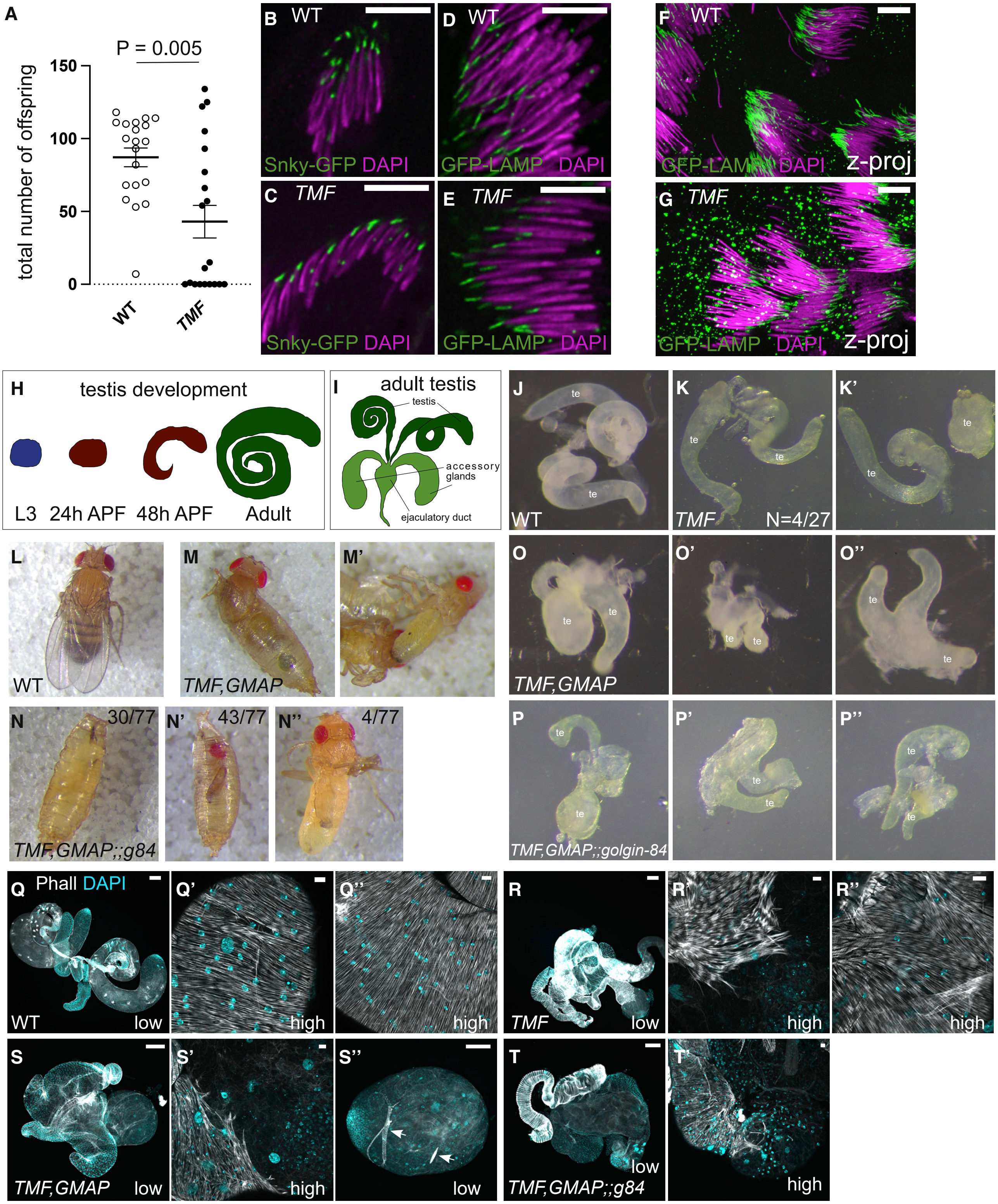

**Figure 5. Phenotypic analyses of intra-Golgi golgin single, double, and triple mutants**

(A) Graph showing the total number of offspring from wild type (WT) and *TMF* mutant males. 20 crosses with two wild-type virgins and one individual male were set up for each genotype (means with error bars showing SEM). Statistical significance determined by unpaired Mann-Whitney test. Data in Data S1.

(B–E) Confocal micrographs of the acrosome markers Snky-GFP (B and C) and GFP-LAMP (D and E) in green and DNA (DAPI) in magenta in developing spermatids of wild type (WT) and *TMF* mutant males. Acrosomes form in *TMF* mutants.

*(legend continued on next page)*

the three intra-Golgi golgins are not essential for the formation of a Golgi stack, consistent with the observation of a polarized distribution of Golgi markers described above (Figure S6).

One of the primary roles of the Golgi is the addition and processing of glycans on glycolipids and glycoproteins. *Drosophila* salivary glands make glue proteins, heavily glycosylated mucins that are stored in granules before release.[51,52] To examine the effect on glycosylation of the golgin mutants, we examined Sgs3, the most abundant of the glue proteins, as perturbation of its glycans is known to affect its gel mobility.[53,54] The *GMAP,golgin-84* and *GMAP,TMF* double mutants caused an increase in the mobility of Sgs3, with an even greater shift seen with the triple mutant, indicating that glycosylation of Sgs3 is impaired (Figure 6F). The lectin *Vicia villosa* agglutinin (VVA) binds the O-linked GalNAc that is initially attached to Sgs3 and other glue proteins before being extended by the addition of galactose to form Galβ1,3GalNAc.[54,55] The GalNAc is attached in the Golgi, and fluorescent VVA labels this compartment in the salivary gland, with this labeling being greatly diminished when the three golgins are removed, again indicating perturbation of Golgi glycosylation processes (Figure 6G).

### TMF acquires GMAP cargo capture function upon loss of endogenous GMAP

The above analysis of the golgin mutants demonstrates that the golgins are partially redundant and collectively essential. This indicates that when one golgin is missing, the presence of a distinct golgin is required to maintain viability. This could be explained by the two golgins simply having near identical roles, but the argument against this is that the individual golgins are not all in the same part of the stack, and moreover, there are different sets of cargo in the vesicles that they capture. To further investigate the basis of redundancy, we performed a golgin mitochondrial relocation assay in the salivary glands of *GMAP* mutants. Strikingly, TMF-mito efficiently captured the cargo Golgin-84 in glands lacking GMAP, despite there being little capture of Golgin-84 by TMF-mito in WT glands (Figures 7A and 7B—compare with Figure 2B). The localization of GFP-TMF in the Golgi stack was not discernibly affected by removal of GMAP (Figures 7C and S7). This capture of Golgin-84 by TMF-mito in the mutant salivary gland is reminiscent of the capture of Golgin-84 by TMF-mito in WT wing imaginal discs (Figure 3B). This indicates that the functional redundancy of the golgins is not due to them simply having overlapping roles, but instead it reflects a plasticity of Golgi organization that allows them to adopt an alternative organization in the absence of a golgin, which is sufficient to maintain cell viability.

## DISCUSSION

Vesicle tethering has emerged as a key step in the process by which transport vesicles are delivered to acceptor organelles and ultimately consumed by SNARE-mediated fusion, but much remains to be resolved about its mechanism and functional significance. In this paper, we have investigated three golgins that share the ability to capture intra-Golgi transport vesicles *in vivo* when tested in mammalian tissue culture cells using a mitochondrial relocation assay. Here, we show that this ectopic relocation approach can also be applied to specific tissues in *Drosophila* and demonstrate that all three of the *Drosophila* orthologs can capture intra-Golgi vesicles. Investigating specific cargo proteins revealed that the content of the captured vesicles varied between the golgins, indicating that they can capture different classes of intra-Golgi carrier. At present, it is not understood in any system what features on the vesicle allow recognition by one golgin and not another, but resolving this would likely help to elucidate how COPI vesicles are able to have multiple roles in the Golgi and ER system.

This relocation approach, using the GAL4-UAS system, is potentially applicable to many other tissues and should thus provide insights into how the organization of Golgi traffic varies depending on cell function. Transport vesicles are small and transient and therefore very hard to visualize and, in the context of a single tissue in an organism, very challenging to purify. However, this ectopic capture approach allows the presence of a particular cargo in a vesicle type to be investigated relatively easily, and indeed it could be applied to other tethers involved in different transport pathways and so reveal the routes by which particular key proteins travel through cells in tissues. In this study, we tested cargo capture by the intra-Golgi golgins in two very different tissues and found that there were clear differences. In particular, Golgin-84 itself, which must recycle to maintain its location in the stack, was efficiently captured by TMF only in wing discs and not in salivary glands. This could reflect Golgin-84 being in the same type of vesicle in each tissue but there being a change in the vesicle recognition processes, or a difference in the trafficking of Golgin-84, such that it is only present in the vesicles recognized by TMF in wing discs and not in salivary glands.

To investigate the functional significance of the three golgins, we generated mutants and found that the loss of any one golgin results in either undetectable or tissue-specific phenotypes. At face value, this result may be surprising given that all three are well conserved in evolution and expressed in most, if not all,

(F and G) As in (B)–(E), but a Z-projection over a larger field of view to show that GFP-LAMP is partially mislocalized in the *TMF* mutant spermatids.
(H and I) Schematics of testis development in the pupa (H) and a mature testis in the adult (I). APF, after puparium formation.
(J and K) Images of testes (te) from wild type (J) and *TMF* mutants (K and K') with morphological defects found in 4/27 cases.
(L–N) Images of flies/pupae of WT (L), *TMF,GMAP* double (M and M'), and *TMF,GMAP;;golgin-84* triple (N–N'') mutants. *TMF,GMAP* double mutants die during or shortly after eclosion, while the majority of *TMF,GMAP;;golgin-84* triple mutants die as pupae.
(O and P) Images of testes from *TMF,GMAP* double (O–O'') and *TMF,GMAP;;golgin-84* triple (P–P'') mutants. These mutants completely fail to form normal testes (te) and exhibit striking morphological defects.
(Q–U) Confocal micrographs of F-actin (phalloidin; gray scale) and DNA (DAPI; cyan) in testes from wild type (WT) and intra-Golgi golgin single, double, and triple mutants. Images were taken at low magnification to show the whole testes (low) or higher magnification to show the actin in the muscle sheath surrounding the testes (high). The muscle sheath is affected by loss of intra-Golgi golgin function. *TMF* single mutants occasionally lack muscles at the tip of the testis and show muscle irregularities. The muscle sheath is strongly affected in *TMF,GMAP* double and *TMF,GMAP;;golgin-84* triple mutants and often fails to cover all of the testes. Occasionally, the muscle is striated instead of smooth (arrows in S''). Scale bars: (B–G) 10 μm. Scale bars: (Q–T) low magnification: 100 μm, high magnification: 10 μm. See also Figure S3.

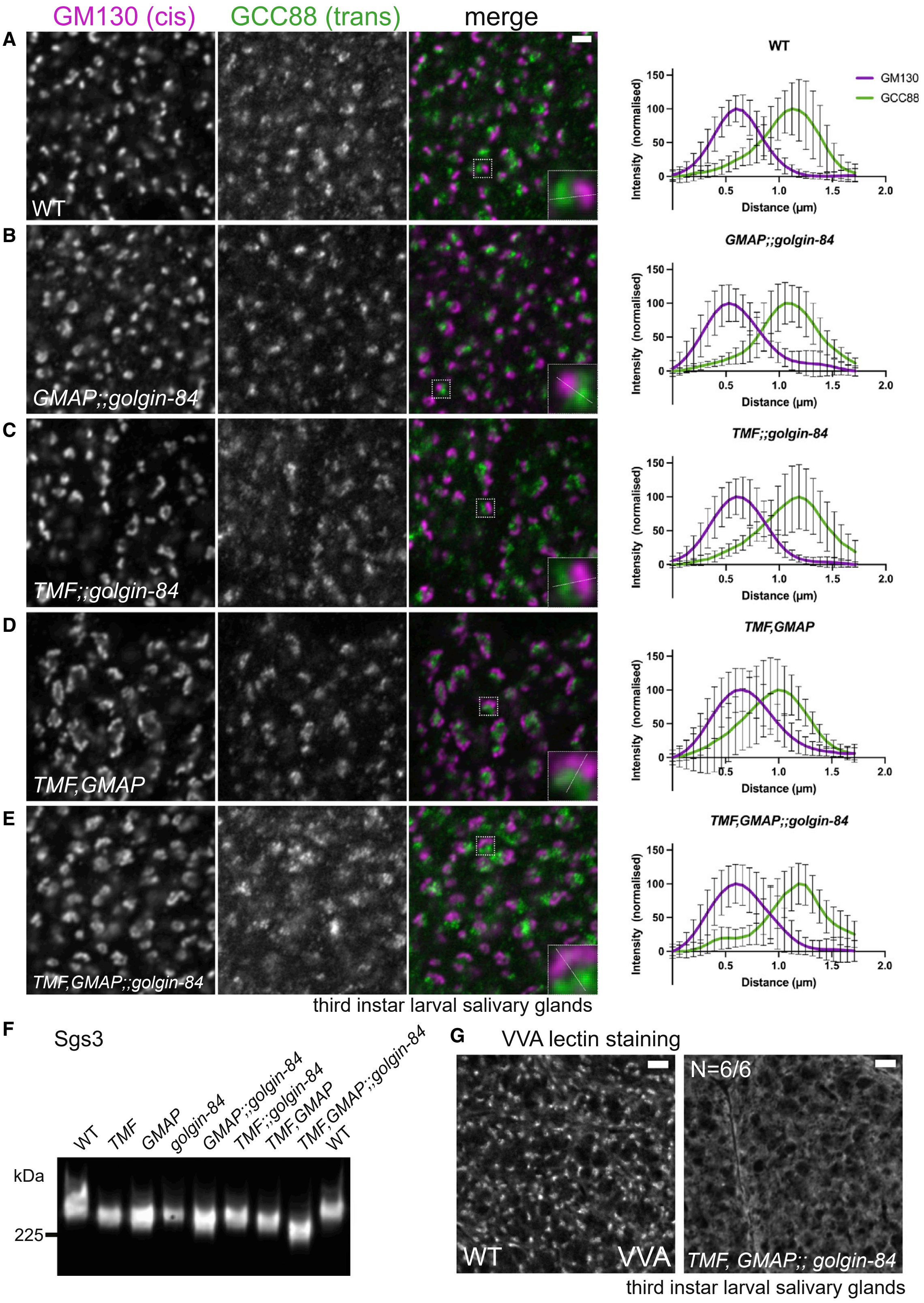


**Figure 6. Intra-Golgi golgin mutants retain Golgi polarity but show strong glycosylation defects**

(A–E) Confocal micrographs of L3 salivary gland cells labeled for the *cis*-Golgi marker GM130 (magenta) and the *trans*-Golgi marker GCC88 (green). Glands prepared from wild type (WT) or the indicated intra-Golgi golgin double and triple mutants. The insets show a higher magnification of the Golgi highlighted in the

*(legend continued on next page)*

tissues, but it is consistent with studies in mice.[28,29,31] However, loss of all three golgins resulted in lethality, a finding that opens a route to structure-function analysis of individual golgins and that also provides some reassurance to those who have been studying these proteins for the last couple of decades. Interestingly, removal of all three golgins did not prevent the formation of polarized Golgi stacks, although more subtle effects would have been hard to detect with thin section EM given the highly pleiomorphic nature of Golgi stacks in *Drosophila* tissues.[35,43,56,57] One possible explanation is that the golgins are sufficient for vesicle capture but other mechanisms could allow some vesicle recycling in their absence. This is similar to the situation with the conserved oligomeric Golgi (COG) complex that has also been proposed to have a role in recycling of intra-Golgi transport vesicles, but when it is deleted from cultured mammalian cells, polarized Golgi stacks are still observed.[58]

Applying the ectopic capture assay to the golgin mutants revealed that although flies that lack GMAP are still viable and fertile, they are not unchanged, as TMF is now able to efficiently capture Golgin-84 in salivary glands, despite this only being observed in the wing disc when GMAP is present. This observation provides an interesting possible explanation for the ability of flies and mice to tolerate the loss of particular golgins. Trafficking routes through the Golgi and endosomal systems are likely to vary greatly between different cell types depending on how much they are secreting, how much recycling of membrane is required, the degree of cell polarity, and if polarized, the relative rates of secretory and endocytic traffic at the different cell surfaces.[59,60] Thus, the entire system must be plastic so that individual organelles can exist and maintain a constant size despite enormous variations in flux rate between different cell types. This plasticity may well be intrinsic to the Golgi/endocytic system to allow ready adaptation to different tissues, as well as adaptation to circumstances where trafficking rates change in response to external cues. It thus seems possible that this plasticity allows cells to compensate for perturbation of a particular component by essentially adapting their endomembrane organization to be more like that of a different tissue. Indeed, lack of cell lethality, or even relatively mild phenotypes in whole animals, arising from the loss of well-conserved proteins has been observed for many other components of traffic in the Golgi-endosomal system, including proteins conserved between metazoans, plants, and fungi. Examples from traffic in the Golgi include GM130, Rab18, SCYL1, and the COG complex and from endosomal traffic include TBC1D23 and the clathrin adaptors AP-3, AP-4, and AP-5.[39,61–66] The conservation of these proteins through evolution indicates that they contribute to fitness. Indeed, in mammals, loss-of-function mutations often cause defects in specialized processes such as bone formation, spermatogenesis, or neurogenesis, where the mutant cells are viable, but it would appear that the degree of plasticity is not enough to accommodate the extreme functional demands of the particular cell type.

Understanding how the endomembrane system functions in the diverse cell types of metazoans is a substantial future challenge. Our work highlights the intra-Golgi golgins as key players in this system, and proffers the use of ectopic vesicle capture as a route to determining the contribution of particular vesicle classes to the functioning of specific cell types.

## STAR★METHODS

Detailed methods are provided in the online version of this paper and include the following:


- KEY RESOURCES TABLE
- RESOURCE AVAILABILITY
  - Lead contact
  - Materials availability
  - Data and code availability
- EXPERIMENTAL MODEL AND SUBJECT DETAILS
  - *Drosophila* stocks
  - *Drosophila* husbandry
- METHOD DETAILS
  - Cloning and ΦC31 transgenesis
  - TEM of salivary glands expressing mito-golgins
  - TEM of salivary glands from golgin mutants
  - Analysis of mitochondrial relocation
  - Fluorescence imaging of testes
  - CRISPR/Cas9-directed tagging and knockouts
  - Immunoblotting
- QUANTIFICATION AND STATISTICAL ANALYSIS


## SUPPLEMENTAL INFORMATION

Supplemental information can be found online at https://doi.org/10.1016/j.cub.2022.08.054.


## ACKNOWLEDGMENTS

We thank Lily Jin for help with microinjections, Kelly Ten Hagen and Liping Zhang for reagents and advice on detecting Sgs3, Catherine Rabouille for reagents and advice on EM of *Drosophila* tissues, Jennifer Richens for helpful discussions, Katja Röper for fly stocks, and Alison Gillingham and Simon Bullock for comments on the manuscript. Funding was from the Medical Research Council, as part of United Kingdom Research and Innovation (also known as UK Research and Innovation), file reference number MC_U105178783.


## AUTHOR CONTRIBUTIONS

S.Y.P., N.M., and S.M. conceptualized and wrote the original draft. S.Y P., N.M., and J.C. investigated and visualized all experimental data. S.Y P. and N.M. were involved in formal analysis. S.M. was involved in funding acquisition and wrote, reviewed, and edited the manuscript.

image. The dotted line illustrates a typical line profile used for quantification, with the graphs showing the normalized means of 10 line profiles of GM130 and GCC88 across the Golgi stack. Error bars, SD. Full data in Data S2.
(F) Immunoblots of total protein extracts from L3 salivary glands from wild type (WT) and intra-Golgi golgin mutants, probed for the heavily glycosylated mucin Sgs3.
(G) Lectin *Vicia villosa* agglutinin (VVA) staining of L3 salivary gland cells of wild type and a mutant lacking GMAP, Golgin-84, and TMF. VVA stains the Golgi in wild-type glands, with specificity confirmed by use of the competing sugar N-acetyl-*D*-galactosamine (GalNAc) at 0.3 M. Scale bars: 2 μm in (A)–(E) and 5 μm in (G).
See also Figures S4–S6.

CellPress
OPEN ACCESS

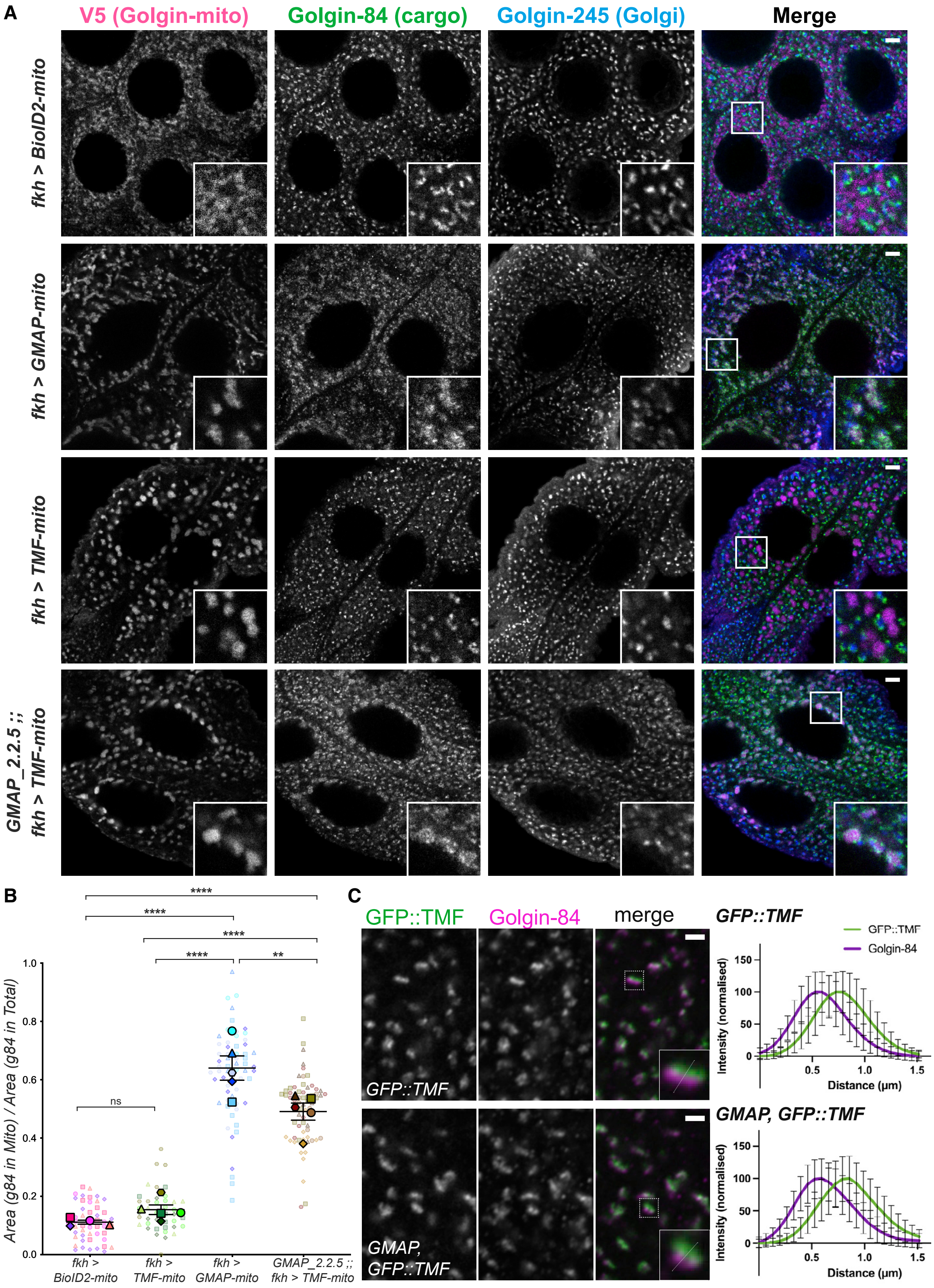


(legend on next page)

## DECLARATION OF INTERESTS

S.M. is a member of the Advisory Board of Current Biology. The authors declare no competing interests.

## INCLUSION AND DIVERSITY

One or more of the authors of this paper self-identifies as a member of the LGBTQ+ community.

Received: November 9, 2021
Revised: June 29, 2022
Accepted: August 18, 2022
Published: September 13, 2022

## REFERENCES

1. Schjoldager, K.T., Narimatsu, Y., Joshi, H.J., and Clausen, H. (2020). Global view of human protein glycosylation pathways and functions. Nat. Rev. Mol. Cell Biol. *21*, 729–749.
2. Sandhoff, R., and Sandhoff, K. (2018). Emerging concepts of ganglioside metabolism. FEBS Lett. *592*, 3835–3864.
3. Moremen, K.W., Tiemeyer, M., and Nairn, A.V. (2012). Vertebrate protein glycosylation: diversity, synthesis and function. Nat. Rev. Mol. Cell Biol. *13*, 448–462.
4. Glick, B.S., and Luini, A. (2011). Models for Golgi traffic: a critical assessment. Cold Spring Harb. Perspect. Biol. *3*, a005215.
5. Munro, S. (2011). What is the Golgi apparatus, and why are we asking? BMC Biol. *9*, 63.
6. Lujan, P., and Campelo, F. (2021). Should I stay or should I go? Golgi membrane spatial organization for protein sorting and retention. Arch. Biochem. Biophys. *707*, 108921.
7. Tu, L., and Banfield, D.K. (2010). Localization of Golgi-resident glycosyltransferases. Cell. Mol. Life Sci. *67*, 29–41.
8. Welch, L.G., and Munro, S. (2019). A tale of short tails, through thick and thin: investigating the sorting mechanisms of Golgi enzymes. FEBS Lett. *593*, 2452–2465.
9. Adolf, F., Rhiel, M., Hessling, B., Gao, Q., Hellwig, A., Béthune, J., and Wieland, F.T. (2019). Proteomic profiling of mammalian COPII and COPI vesicles. Cell Rep. *26*, 250–265.e5.
10. Pantazopoulou, A., and Glick, B.S. (2019). A kinetic view of membrane traffic pathways can transcend the classical view of Golgi compartments. Front. Cell Dev. Biol. *7*, 153.
11. Eckert, E.S.P., Reckmann, I., Hellwig, A., Röhling, S., El-Battari, A., Wieland, F.T., and Popoff, V. (2014). Golgi phosphoprotein 3 triggers signal-mediated incorporation of glycosyltransferases into coatomer-coated (COPI) vesicles. J. Biol. Chem. *289*, 31319–31329.
12. Witkos, T.M., and Lowe, M. (2017). Recognition and tethering of transport vesicles at the Golgi apparatus. Curr. Opin. Cell Biol. *47*, 16–23.
13. Yu, I.M., and Hughson, F.M. (2010). Tethering factors as organizers of intracellular vesicular traffic. Annu. Rev. Cell Dev. Biol. *26*, 137–156.
14. Gillingham, A.K., and Munro, S. (2016). Finding the Golgi: golgin coiled-coil proteins show the way. Trends Cell Biol. *26*, 399–408.
15. Witkos, T.M., and Lowe, M. (2015). The golgin family of coiled-coil tethering proteins. Front. Cell Dev. Biol. *3*, 86.
16. Muschalik, N., and Munro, S. (2018). Golgins. Curr. Biol. *28*, R374–R376.
17. Wong, M., and Munro, S. (2014). Membrane trafficking. The specificity of vesicle traffic to the Golgi is encoded in the golgin coiled-coil proteins. Science *346*, 1256898.
18. Sato, K., Roboti, P., Mironov, A.A., and Lowe, M. (2015). Coupling of vesicle tethering and Rab binding is required for in vivo functionality of the golgin GMAP-210. Mol. Biol. Cell *26*, 537–553.
19. Shin, J.J.H., Crook, O.M., Borgeaud, A.C., Cattin-Ortolá, J., Peak-Chew, S.Y., Breckels, L.M., Gillingham, A.K., Chadwick, J., Lilley, K.S., and Munro, S. (2020). Spatial proteomics defines the content of trafficking vesicles captured by golgin tethers. Nat. Commun. *11*, 5987.
20. Gillingham, A.K., Tong, A.H.Y., Boone, C., and Munro, S. (2004). The GTPase Arf1p and the ER to Golgi cargo receptor Erv14p cooperate to recruit the golgin Rud3p to the cis-Golgi. J. Cell Biol. *167*, 281–292.
21. Bascom, R.A., Srinivasan, S., and Nussbaum, R.L. (1999). Identification and characterization of golgin-84, a novel Golgi integral membrane protein with a cytoplasmic coiled-coil domain. J. Biol. Chem. *274*, 2953–2962.
22. Mori, K., and Kato, H. (2002). A putative nuclear receptor coactivator (TMF/ARA160) associates with hbrm/hSNF2 alpha and BRG-1/hSNF2 beta and localizes in the Golgi apparatus. FEBS Lett. *520*, 127–132.
23. Diao, A., Rahman, D., Pappin, D.J.C., Lucocq, J., and Lowe, M. (2003). The coiled-coil membrane protein golgin-84 is a novel rab effector required for Golgi ribbon formation. J. Cell Biol. *160*, 201–212.
24. Fridmann-Sirkis, Y., Siniossoglou, S., and Pelham, H.R.B. (2004). TMF is a golgin that binds Rab6 and influences Golgi morphology. BMC Cell Biol. *5*, 18.
25. Rios, R.M., Tassin, A.M., Celati, C., Antony, C., Boissier, M.C., Homberg, J.C., and Bornens, M. (1994). A peripheral protein associated with the cis-Golgi network redistributes in the intermediate compartment upon brefeldin A treatment. J. Cell Biol. *125*, 997–1013.
26. Wong, M., Gillingham, A.K., and Munro, S. (2017). The golgin coiled-coil proteins capture different types of transport carriers via distinct N-terminal motifs. BMC Biol. *15*, 3.
27. Lowe, M. (2019). The physiological functions of the golgin vesicle tethering proteins. Front. Cell Dev. Biol. *7*, 94.
28. McGee, L.J., Jiang, A.L., and Lan, Y. (2017). Golga5 is dispensable for mouse embryonic development and postnatal survival. Genesis *55*, e23039.
29. Lerer-Goldshtein, T., Bel, S., Shpungin, S., Pery, E., Motro, B., Goldstein, R.S., Bar-Sheshet, S.I., Breitbart, H., and Nir, U. (2010). TMF/ARA160: a key regulator of sperm development. Dev. Biol. *348*, 12–21.

**Figure 7. TMF-mito acquires Golgin-84 vesicle capture function in GMAP mutant larval salivary glands**
(A) Confocal micrographs of L3 salivary glands expressing golgin-mito constructs, or the BioID2-mito negative control, and labeled for the V5 tag (magenta), endogenous Golgin-84 (green), and *trans*-Golgi marker Golgin-245 (blue). Glands are from wild type (first three rows) and *GMAP* mutants (bottom row). Insets are zooms of the boxed regions in the merge. Scale bars: 5 μm.
(B) Quantification of the mitochondrial relocalization of Golgin-84 in (A), using the ratio between the area of cargo in mitochondria and total area of cargo. Cell-by-cell ratios are plotted as small, partially transparent, points; larval ratios (averaged cell-by-cell ratios) are plotted as large opaque points. Points are shaped and colored according to larval replicate, with the format of cell-by-cell points matching that of their corresponding larval means. The mean larval ratio (line) and SEM (bars) are plotted for each genotype. Data analyzed by one-way nested ANOVA using Tukey's multiple comparisons, ns, not significant, $^{**}p \leq 0.01$ and $^{****}p \leq 0.0001$. n = 5 larvae (4 for BioID2-mito), with 6–19 cells segmented per larvae. Full data in Data S1.
(C) Confocal micrographs of L3 salivary gland cells prepared from *GFP::TMF* control or *GMAP,GFP::TMF* mutants and labeled for GFP::TMF (green) and the medial-Golgi marker Golgin-84 (magenta). Insets show a zoom of the Golgi highlighted in the image. The dotted line illustrates a typical line profile used for quantification, with the graphs showing the normalized means of 15–30 line profiles of the marker proteins across the Golgi stack. Error bars, SD. Scale bars: 2 μm, full data in Data S2. See also Figure S7.

30. Yamaguchi, H., Meyer, M.D., He, L., Senavirathna, L., Pan, S., and Komatsu, Y. (2021). The molecular complex of ciliary and golgin protein is crucial for skull development. Development *148*, dev199559.

31. Smits, P., Bolton, A.D., Funari, V., Hong, M., Boyden, E.D., Lu, L., Manning, D.K., Dwyer, N.D., Moran, J.L., Prysak, M., et al. (2010). Lethal skeletal dysplasia in mice and humans lacking the golgin GMAP-210. N. Engl. J. Med. *362*, 206–216.

32. Townley, A.K., Feng, Y., Schmidt, K., Carter, D.A., Porter, R., Verkade, P., and Stephens, D.J. (2008). Efficient coupling of Sec23-Sec24 to Sec13-Sec31 drives COPII-dependent collagen secretion and is essential for normal craniofacial development. J. Cell Sci. *121*, 3025–3034.

33. Unlu, G., Levic, D.S., Melville, D.B., and Knapik, E.W. (2014). Trafficking mechanisms of extracellular matrix macromolecules: insights from vertebrate development and human diseases. Int. J. Biochem. Cell Biol. *47*, 57–67.

34. Hou, N., Yang, Y., Scott, I.C., and Lou, X. (2017). The Sec domain protein Scfd1 facilitates trafficking of ECM components during chondrogenesis. Dev. Biol. *421*, 8–15.

35. Kondylis, V., and Rabouille, C. (2009). The Golgi apparatus: lessons from *Drosophila*. FEBS Lett. *583*, 3827–3838.

36. Ke, H., Feng, Z., Liu, M., Sun, T., Dai, J., Ma, M., Liu, L.-P., Ni, J.-Q., and Pastor-Pareja, J.C. (2018). Collagen secretion screening in *Drosophila* supports a common secretory machinery and multiple Rab requirements. J. Genet. Genomics *45*, 299–313.

37. Satoh, T., Nakamura, Y., and Satoh, A.K. (2016). The roles of Syx5 in Golgi morphology and rhodopsin transport in *Drosophila* photoreceptors. Biol. Open *5*, 1420–1430.

38. Ma, C.-I.J., Yang, Y., Kim, T., Chen, C.H., Polevoy, G., Vissa, M., Burgess, J., and Brill, J.A. (2020). An early endosome-derived retrograde trafficking pathway promotes secretory granule maturation. J. Cell Biol. *219*, e201808017.

39. Belloni, G., Sechi, S., Riparbelli, M.G., Fuller, M.T., Callaini, G., and Giansanti, M.G. (2012). Mutations in Cog7 affect Golgi structure, meiotic cytokinesis and sperm development during *Drosophila* spermatogenesis. J. Cell Sci. *125*, 5441–5452.

40. Wainman, A., Giansanti, M.G., Goldberg, M.L., and Gatti, M. (2012). The *Drosophila* RZZ complex – roles in membrane trafficking and cytokinesis. J. Cell Sci. *125*, 4014–4025.

41. Munro, S. (2011). The golgin coiled-coil proteins of the Golgi apparatus. Cold Spring Harb. Perspect. Biol. *3*, a005256.

42. Riedel, F., Gillingham, A.K., Rosa-Ferreira, C., Galindo, A., and Munro, S. (2016). An antibody toolkit for the study of membrane traffic in *Drosophila melanogaster*. Biol. Open *5*, 987–992.

43. Friggi-Grelin, F., Rabouille, C., and Therond, P. (2006). The cis-Golgi *Drosophila* GMAP has a role in anterograde transport and Golgi organization in vivo, similar to its mammalian ortholog in tissue culture cells. Eur. J. Cell Biol. *85*, 1155–1166.

44. Yamamoto-Hino, M., Abe, M., Shibano, T., Setoguchi, Y., Awano, W., Ueda, R., Okano, H., and Goto, S. (2012). Cisterna-specific localization of glycosylation-related proteins to the Golgi apparatus. Cell Struct. Funct. *37*, 55–63.

45. Sisson, J.C., Field, C., Ventura, R., Royou, A., and Sullivan, W. (2000). Lava lamp, a novel peripheral Golgi protein, is required for *Drosophila melanogaster* cellularization. J. Cell Biol. *151*, 905–918.

46. Tuladhar, R., Yeu, Y., Tyler Piazza, J., Tan, Z., Rene Clemenceau, J., Wu, X., Barrett, Q., Herbert, J., Mathews, D.H., Kim, J., et al. (2019). CRISPR-Cas9-based mutagenesis frequently provokes on-target mRNA misregulation. Nat. Commun. *10*, 4056.

47. Han, F., Liu, C., Zhang, L., Chen, M., Zhou, Y., Qin, Y., Wang, Y., Chen, M., Duo, S., Cui, X., et al. (2017). Globozoospermia and lack of acrosome formation in GM130-deficient mice. Cell Death Dis. *8*, e2532.

48. Bentson, L.F., Agbor, V.A., Agbor, L.N., Lopez, A.C., Nfonsam, L.E., Bornstein, S.S., Handel, M.A., and Linder, C.C. (2013). New point mutation in Golga3 causes multiple defects in spermatogenesis. Andrology *1*, 440–450.

49. Rothenbusch-Fender, S., Fritzen, K., Bischoff, M.C., Buttgereit, D., Oenel, S.F., and Renkawitz-Pohl, R. (2017). Myotube migration to cover and shape the testis of *Drosophila* depends on Heartless, Cadherin/Catenin, and myosin II. Biol. Open *6*, 1876–1888.

50. Susic-Jung, L., Hornbruch-Freitag, C., Kuckwa, J., Rexer, K.-H., Lammel, U., and Renkawitz-Pohl, R. (2012). Multinucleated smooth muscles and mononucleated as well as multinucleated striated muscles develop during establishment of the male reproductive organs of *Drosophila melanogaster*. Dev. Biol. *370*, 86–97.

51. Biyasheva, A., Do, T.V., Lu, Y., Vaskova, M., and Andres, A.J. (2001). Glue secretion in the *Drosophila* salivary gland: a model for steroid-regulated exocytosis. Dev. Biol. *231*, 234–251.

52. Tran, D.T., and Ten Hagen, K.G. (2013). Mucin-type O-glycosylation during development. J. Biol. Chem. *288*, 6921–6929.

53. Reynolds, H.M., Zhang, L., Tran, D.T., and Ten Hagen, K.G. (2019). Tango1 coordinates the formation of endoplasmic reticulum/Golgi docking sites to mediate secretory granule formation. J. Biol. Chem. *294*, 19498–19510.

54. Ji, S., Samara, N.L., Revoredo, L., Zhang, L., Tran, D.T., Muirhead, K., Tabak, L.A., and Hagen, ten, K.G. (2018). A molecular switch orchestrates enzyme specificity and secretory granule morphology. Nat. Commun. *9*, 3508.

55. Tian, E., and Ten Hagen, K.G. (2007). O-linked glycan expression during *Drosophila* development. Glycobiology *17*, 820–827.

56. Zuber, C., Fan, J.Y., Guhl, B., Parodi, A., Fessler, J.H., Parker, C., and Roth, J. (2001). Immunolocalization of UDP-glucose:glycoprotein glucosyltransferase indicates involvement of pre-Golgi intermediates in protein quality control. Proc. Natl. Acad. Sci. USA *98*, 10710–10715.

57. Lane, N.J., Carter, Y.R., and Ashburner, M. (1972). Puffs and salivary gland function: the fine structure of the larval and prepupal salivary glands of *Drosophila melanogaster*. Wilhelm Roux Arch. Entwickl. Mech. Org. *169*, 216–238.

58. Ungar, D., Oka, T., Brittle, E.E., Vasile, E., Lupashin, V.V., Chatterton, J.E., Heuser, J.E., Krieger, M., and Waters, M.G. (2002). Characterization of a mammalian Golgi-localized protein complex, COG, that is required for normal Golgi morphology and function. J. Cell Biol. *157*, 405–415.

59. Rodriguez-Boulan, E., Kreitzer, G., and Müsch, A. (2005). Organization of vesicular trafficking in epithelia. Nat. Rev. Mol. Cell Biol. *6*, 233–247.

60. Apodaca, G., and Brown, W.J. (2014). Membrane traffic research: challenges for the next decade. Front. Cell Dev. Biol. *2*, 52.

61. Bem, D., Yoshimura, S., Nunes-Bastos, R., Bond, F.C., Kurian, M.A., Rahman, F., Handley, M.T., Hadzhiev, Y., Masood, I., Straatman-Iwanowska, A.A., et al. (2011). Loss-of-function mutations in RAB18 cause Warburg micro syndrome. Am. J. Hum. Genet. *88*, 499–507.

62. Hellicar, J., Stevenson, N.L., Stephens, D.J., and Lowe, M. (2022). Supply chain logistics – the role of the Golgi complex in extracellular matrix production and maintenance. J. Cell Sci. *135*, jcs258879.

63. Marin-Valencia, I., Gerondopoulos, A., Zaki, M.S., Ben-Omran, T., Almureikhi, M., Demir, E., Guemez-Gamboa, A., Gregor, A., Issa, M.Y., Appelhof, B., et al. (2017). Homozygous mutations in TBC1D23 lead to a non-degenerative form of pontocerebellar hypoplasia. Am. J. Hum. Genet. *101*, 441–450.

64. Schmidt, W.M., Kraus, C., Höger, H., Hochmeister, S., Oberndorfer, F., Branka, M., Bingemann, S., Lassmann, H., Müller, M., Macedo-Souza, L.I., et al. (2007). Mutation in the Scyl1 gene encoding amino-terminal kinase-like protein causes a recessive form of spinocerebellar neurodegeneration. EMBO Rep. *8*, 691–697.

65. Liu, C., Mei, M., Li, Q., Roboti, P., Pang, Q., Ying, Z., Gao, F., Lowe, M., and Bao, S. (2017). Loss of the golgin GM130 causes Golgi disruption, Purkinje neuron loss, and ataxia in mice. Proc. Natl. Acad. Sci. USA *114*, 346–351.

66. Sanger, A., Hirst, J., Davies, A.K., and Robinson, M.S. (2019). Adaptor protein complexes and disease at a glance. J. Cell Sci. *132*, jcs222992.

67. Sinka, R., Gillingham, A.K., Kondylis, V., and Munro, S. (2008). Golgi coiled-coil proteins contain multiple binding sites for Rab family G proteins. J. Cell Biol. *183*, 607–615.

68. Ivan, V., de Voer, G., Xanthakis, D., Spoorendonk, K.M., Kondylis, V., and Rabouille, C. (2008). *Drosophila* Sec16 mediates the biogenesis of tER sites upstream of Sar1 through an arginine-rich motif. Mol. Biol. Cell *19*, 4352–4365.

69. Torres, I.L., Rosa-Ferreira, C., and Munro, S. (2014). The Arf family G protein Arl1 is required for secretory granule biogenesis in *Drosophila*. J. Cell Sci. *127*, 2151–2160.

70. Port, F., Chen, H.-M., Lee, T., and Bullock, S.L. (2014). Optimized CRISPR/Cas tools for efficient germline and somatic genome engineering in *Drosophila*. Proc. Natl. Acad. Sci. USA *111*, E2967–E2976.

71. Wilson, K.L., Fitch, K.R., Bafus, B.T., and Wakimoto, B.T. (2006). Sperm plasma membrane breakdown during *Drosophila* fertilization requires sneaky, an acrosomal membrane protein. Development *133*, 4871–4879.

72. Pulipparacharuvil, S., Akbar, M.A., Ray, S., Sevrioukov, E.A., Haberman, A.S., Rohrer, J., and Krämer, H. (2005). *Drosophila* Vps16A is required for trafficking to lysosomes and biogenesis of pigment granules. J. Cell Sci. *118*, 3663–3673.

73. Schindelin, J., Arganda-Carreras, I., Frise, E., Kaynig, V., Longair, M., Pietzsch, T., Preibisch, S., Rueden, C., Saalfeld, S., Schmid, B., et al. (2012). Fiji: an open-source platform for biological-image analysis. Nat. Methods *9*, 676–682.

74. Gratz, S.J., Ukken, F.P., Rubinstein, C.D., Thiede, G., Donohue, L.K., Cummings, A.M., and O'Connor-Giles, K.M. (2014). Highly specific and efficient CRISPR/Cas9-catalyzed homology-directed repair in *Drosophila*. Genetics *196*, 961–971.

75. Lowe, N., Rees, J.S., Roote, J., Ryder, E., Armean, I.M., Johnson, G., Drummond, E., Spriggs, H., Drummond, J., Magbanua, J.P., et al. (2014). Analysis of the expression patterns, subcellular localisations and interaction partners of *Drosophila* proteins using a pigP protein trap library. Development *141*, 3994–4005.

76. Bischof, J., Maeda, R.K., Hediger, M., Karch, F., and Basler, K. (2007). An optimized transgenesis system for *Drosophila* using germ-line-specific phiC31 integrases. Proc. Natl. Acad. Sci. USA *104*, 3312–3317.

77. Burgess, J., Jauregui, M., Tan, J., Rollins, J., Lallet, S., Leventis, P.A., Boulianne, G.L., Chang, H.C., Le Borgne, R., Krämer, H., and Brill, J.A. (2011). AP-1 and clathrin are essential for secretory granule biogenesis in *Drosophila*. Mol. Biol. Cell *22*, 2094–2105.

78. Lavancier, F., Pécot, T., Zengzhen, L., and Kervrann, C. (2020). Testing independence between two random sets for the analysis of colocalization in bioimaging. Biometrics *76*, 36–46.

79. Lord, S.J., Velle, K.B., Mullins, R.D., and Fritz-Laylin, L.K. (2020). SuperPlots: communicating reproducibility and variability in cell biology. J. Cell Biol. *219*, e202001064.

## STAR★METHODS

### KEY RESOURCES TABLE

| REAGENT or RESOURCE | SOURCE | IDENTIFIER |
|---|---|---|
| Antibodies | | |
| Mouse anti-V5 | ThermoFisher Scientific | R960-25; RRID:AB_2556564 |
| Rabbit anti-V5 | Cell Signaling Technology | 13202; RRID:AB_2687461 |
| Chicken anti-V5 | Bethyl | A190-118A; RRID:AB_66741 |
| Rabbit anti-βactin | Abcam | ab8227; RRID:AB_2305186 |
| Mouse anti-Golgin-84 | DSHB | Golgin84 12-1; RRID:AB_2722113 |
| Goat anti-GMAP | DSHB | GMAP; RRID:AB_2618259 |
| Goat anti-Golgin-245 | DSHB | Golgin245; RRID:AB_2569587 |
| Rabbit anti-GM130 | Abcam | ab30637; RRID:AB_732675 |
| Guinea pig anti-GCC88 | Sinka et al.[67] | N/A |
| Rabbit anti-Lava lamp | Sisson, et al.[45] | N/A |
| Rabbit anti-Sec16 | Ivan et al.[68] | N/A |
| Chicken anti-Arl1 | Torres et al.[69] | N/A |
| Rat anti-Glg1 | Yamamoto-Hino et al.[44] | N/A |
| Rabbit anti-Sgs3 | Reynolds et al.[53] | N/A |
| Rabbit anti-Syntaxin 16 | Abcam | ab32340; RRID:AB_778213 |
| Mouse anti-Complex V α (15H4C4) | ThermoFisher Scientific | 43-9800; RRID:AB_2533548 |
| Donkey anti-Mouse IgG (Alexa 488) | ThermoFisher Scientific | A21202; RRID:AB_141607 |
| Donkey anti-Rat IgG (Alexa 488) | ThermoFisher Scientific | A21208; RRID:AB_2535794 |
| Donkey anti-Rabbit IgG (Alexa 555) | ThermoFisher Scientific | A31572; RRID:AB_162543 |
| Donkey anti-Goat IgG (Alexa 555) | ThermoFisher Scientific | A21432; RRID:AB_2535853 |
| Donkey anti-Rabbit IgG (Alexa 647) | ThermoFisher Scientific | A31573; RRID:AB_2536183 |
| Donkey anti-Mouse IgG (Alexa 647) | ThermoFisher Scientific | A31571; RRID:AB_162542 |
| Donkey anti-Rat IgG (Alexa 647) | ThermoFisher Scientific | A48272; RRID:AB_2893138 |
| Donkey anti-chicken IgY (Cy3) | Jackson ImmunoResearch | 703-165-155; RRID:AB_2340363 |
| Donkey anti-chicken IgY (Cy5) | Jackson ImmunoResearch | 703-175-155; RRID:AB_2340365 |
| Sheep anti-Mouse IgG (HRP) | Amersham | NA931; RRID:AB_772210 |
| Donkey anti-Rabbit IgG (HRP) | Amersham | NA934; RRID:AB_772206 |
| Mouse anti-Goat IgG (HRP) | Santa Cruz Biotechnology | Sc-2354; RRID:AB_628490 |
| Chemicals, peptides, and recombinant proteins | | |
| GFP booster-Atto488 | Chromotek | gba488; RRID:AB_2631386 |
| GFP booster-Atto647N | Chromotek | gba647n; RRID:AB_2629215 |
| Vicia villosa lectin (VVA, fluorescein) | Vector | FL-1231; RRID:AB_2336856 |
| CY212 epoxy resin | Agar Scientific | AGR1030 |
| Aclar film | Agar Scientific | AGL4458 |
| Vectashield mounting medium with DAPI | Vector Laboratories | H-1200 |
| $H_2O_2$-urea adduct | Sigma-Aldrich | 95314 |
| DAB with cobalt enhancer | Sigma-Aldrich | D0426 |
| Restore PLUS Western Blot Stripping Buffer | ThermoFisher Scientific | 46430 |
| MicroLYSIS-Plus DNA release buffer | Clent Life Science | 2MLP |
| Deposited data | | |
| Confocal micrographs used for quantifying mitochondrial relocation | This paper; Mendeley Data | https://doi.org/10.17632/yr87kr8wbz.1 |

(*Continued on next page*)

*Continued*

| REAGENT or RESOURCE | SOURCE | IDENTIFIER |
|---|---|---|
| Experimental models: Organisms/strains | | |
| D. mel: GFP::TMF | This study | N/A |
| D. mel: αManII-IIa::GFP | This study | N/A |
| D. mel: GMAP_2.2.5 | This study | N/A |
| D. mel: TMF_Δ19 | This study | N/A |
| D. mel: golgin84_ Δ13.3 | This study | N/A |
| D. mel: UASt-BioID2-mito (II) | This study | N/A |
| D. mel: UASt-BioID2-mito (III) | This study | N/A |
| D. mel: UASt-g245-mito (II) | This study | N/A |
| D. mel: UASt-g245-mito (III) | This study | N/A |
| D. mel: UASt-TMF-mito (II) | This study | N/A |
| D. mel: UASt-TMF-mito (III) | This study | N/A |
| D. mel: UASt-g84-mito (II) | This study | N/A |
| D. mel: UASt-g84-mito (III) | This study | N/A |
| D. mel: UASt-GMAP-mito (II) | This study | N/A |
| D. mel: UASt-GMAP-mito (III) | This study | N/A |
| D. mel: CFD2 | Port et al.[70] | N/A |
| D. mel: TH_attP2 | Port et al.[70] | N/A |
| D. mel: Snky-GFP | Wilson et al.[71] | N/A |
| D. mel: GFP-LAMP | Pulipparacharuvil et al.[72] | N/A |
| D. mel: fkh-GAL4 | Bloomington Drosophila Stock Centre | 78060; RRID:BDSC_78060 |
| D. mel: en-GAL4 | Bloomington Drosophila Stock Centre | 1973; RRID:BDSC_1973 |
| D. mel: YFP::Pgant9 | Kyoto Stock Centre | 115535; RRID:DGGR_115535 |
| Recombinant DNA | | |
| Plasmid MCS-BioID2-HA | Addgene | 74224; RRID:Addgene_74224 |
| cDNA FI19713 | Drosophila Genomics Resource Centre. | 1660994; RRID:DGRC_1660994 |
| cDNA RE70149 | Drosophila Genomics Resource Centre. | 9965; RRID:DGRC_9965 |
| cDNA SD07366 | Drosophila Genomics Resource Centre. | 5395; RRID:DGRC_5395 |
| cDNA SD05887 | Drosophila Genomics Resource Centre. | 6640; RRID:DGRC_6640 |
| Plasmid pCFD3 | Addgene | 49408; RRID:Addgene_49408 |
| Software and algorithms | | |
| Fiji | Schindelin et al.[73] | https://fiji.sc/ |
| GraphPad Prism 8 | GraphPad Software | https://www.graphpad.com |
| CRISPR Optimal Target Finder | Gratz et al.[74] | http://targetfinder.flycrispr.neuro.brown.edu/) |
| NIS Elements AR 5.02.03 | Nikon Instruments Inc | N/A |

## RESOURCE AVAILABILITY

### Lead contact

Further information and requests for resources and reagents should be directed to the lead contact, Sean Munro (sean@mrc-lmb.cam.ac.uk).

### Materials availability

The *Drosophila* lines and plasmids generated in this study are available from the lead contact upon request.

### Data and code availability

- Original data from confocal figures used for quantifying relocation to mitochondria have deposited here: Mendeley Data: https://doi.org/10.17632/yr87kr8wbz.1, as listed in the key resources table. All other data reported in this paper will be shared by the lead contact upon reasonable request.

- This paper does not report original code.
- Any additional information required to reanalyze the data reported in this paper is available from the lead contact upon request.

## EXPERIMENTAL MODEL AND SUBJECT DETAILS

### *Drosophila* stocks

The following fly stocks were used in this study: CFD2 and TH_attP2,[70] Snky-GFP,[71] GFP-LAMP,[72] GFP::TMF (this study, see below) αManII::GFP (this study, see below), *GMAP_2.2.5*, *TMF_Δ19*, *golgin-84_ Δ13.3* (all three this study, see below), fkh-GAL4 (BDSC 78060), en-GAL4 (BDSC 1973), and YFP::pgant9 ([75], Kyoto Stock Centre 115535). The following UAS-X-mito stocks were generated (this study, see ΦC31 transgenesis): UASt-BioID2-mito (II), UASt-BioID2-mito (III), UASt-g245-mito (II), UASt-g245-mito (III), UASt-TMF-mito (II), UASt-TMF-mito (III), UASt-g84-mito (II), UASt-g84-mito (III), UASt-GMAP-mito (II), UASt-GMAP-mito (III).

### *Drosophila* husbandry

All fly stocks and crosses were kept at 25°C using a 12-h-light-12-h-dark cycle, unless stated otherwise. All flies were kept using the same food, and crosses were knocked over onto new food twice a week. OregonR was used as wild type unless stated otherwise. For golgin mutants, both females and males were used for experiments except for *TMF*,*GMAP* double and *TMF*,*GMAP*;;*golgin-84* triple mutants in which case only males could be used for analysis due to the mutant phenotype. For mitochondrial relocation experiments, both males and females were analysed for all genotypes, except for *GMAP_2.2.5;; fkh > UAS-TMF-mito* in which only males hemizygous for *GMAP_2.2.5* allele were collected as GMAP is on the X chromosome. To assay male fertility in *TMF* mutants, 20 crosses with a single male and two wild-type virgins were set up for each genotype. The total number of offspring was counted for all crosses and the mean +/- SEM was plotted for each genotype.

## METHOD DETAILS

### Cloning and ΦC31 transgenesis

*Drosophila* TMF, Golgin-84, and GMAP were C-terminally truncated based on previous mitochondrially targeted human orthologues.[17] The sequences of each GolginΔC are as follows: TMF (CG4557, FBgn0029912), residues 1-609; Golgin-84 (Golgin84, FBgn0039188), residues 1-471; GMAP (GMAP, FBgn0027287), residues 1-1194; and Golgin-245 (Golgin245, FBgn0034854), residues 1-1428. EcoRI-GolginΔC-NotI regions were amplified from cDNA clones FI19713, RE70149, SD07366, and SD05887 (Drosophila Genomics Resource Centre), and inserted into EcoRI/NotI digested pUAST_attB-V5-MAO vector (this study). The negative control BioID2-mito construct was generated by amplifying BioID2-EagI-GAGAGA from MCS-BioID2-HA (Addgene) and V5-MAO from pUAST_attB-V5-MAO, followed by NEB HiFi assembly (NEB) of the fragments with EcoRI/XhoI digested pUAST_attB vector. pUAST_attB- GolginΔC-V5-MAO plasmids were injected (L. Jin, MRC-LMB; and the Department of Genetics Fly Facility, University of Cambridge) into *vas*-ΦC31; attP40 or *nos*-ΦC31;; attP2 embryos to generate UASt-golgin-mito (II) and (III) transgenic lines.[76]

### TEM of salivary glands expressing mito-golgins

Salivary glands from wandering L3 larvae were dissected in Ringer solution at room temperature (RT) and incubated in fixative (2.5% glutaraldehyde, 2% paraformaldehyde in 0.1 M sodium cacodylate, 5 mM $CaCl_2$, 10 mM $MgCl_2$) for 4 hours at RT and then incubated in fresh fixative overnight at 4°C. Fixative was removed and unreacted aldehydes were quenched by washing glands in 0.1 M sodium cacodylate, 2 mM $CaCl_2$, 20 mM glycine for 3 x 5min and then 3 x 30 min at RT. Glands were washed again for 3 x 5 mins in 0.1 M phosphate at RT, then stained on ice for 30 mins with $H_2O_2$, diaminobenzidine and cobalt (Sigma-Aldrich), on a shaker and covered with foil. Discs were then rinsed in 0.1 M phosphate buffer for 3 x 5mins at RT and fixed for a second time with EM fixative for a further 16 hours at RT on a shaker. They were then washed with 0.1 M phosphate buffer for 3 x 5 mins then 3 x 30 mins and post-fixed with reduced osmium (1% OsO4, 1.5% K3[Fe(CN6)]) for 1 hour at 4°C covered with foil. Discs were rinsed in ddH2O for 5 x 10 mins then post-fixed again in 0.5% uranyl acetate in ddH2O for 1 hour at 4°C in the dark. Sample dehydration was via incubation in 70% ethanol overnight at 4°C, followed by further dehydration at RT in 90% for 15 min, 96% for 15 minutes, and 100% ethanol for 3 x 30 min. Glands were incubated in 100% polypropylene oxide (PPO) for 2 x 10 min, then infiltrated with a series of 1:3, 1:1, and 3:1 mixtures of Araldite CY212 epoxy resin (Agar Scientific) and PPO for 1 hour each at RT, before incubation in 100% resin for 2-3 hours. Resin was exchanged multiple times throughout 1 week, before sample embedding in CY212 resin between Aclar sheets (Agar Scientific). Samples were polymerised for 24-48 hours at 50-60°C and then sliced into 70 nm sections on a Reichert Ultracut E microtome. Sections were mounted on carbon coated copper grids, stained in 2% uranyl acetate in ddH2O for 30 minutes, and imaged on FEI Tecnai G-Spirit transmission electron microscopes at 80kV at RT.

### TEM of salivary glands from golgin mutants

Salivary glands from L3 wandering larvae of the desired genotype were dissected in 2.5% glutaraldehyde, 2% formaldehyde and 2% paraformaldehyde in 0.1 M phosphate buffer heated to 37°C and fixed for 1 hour at 37°C followed by o/n fixation (RT). The following day, glands were washed in RT 0.1 M phosphate buffer for 3 x 5 minutes followed by 3 x 30 minutes. Double post-fixation of the lipid

membranes was then carried out by initially fixing in 1% osmium tetroxide and 1.5% potassium ferrocyanide in 0.1 M phosphate buffer for 2 hours in the dark at 4°C. Washes in double distilled (DD) water followed for 3x5 minutes then 3 x 30 minutes before using 0.5% uranyl acetate in DD water for 1 hour in the dark at RT for the second fixation step. Glands were washed in DD water again for 3 x 5 minutes then 3 x30 minutes before a dehydration step using graded ethanol in the following sequence: 70% overnight at 4°C, 90% for 15 minutes at RT, 96% for 15 minutes at RT and 100% for 3 x 5 minutes then 3 x 30 minutes. To prepare the glands for infiltration of CY212 epoxy resin (Agar Scientific), 100% propylene oxide (PPO) was then incubated for 2 x 10 minutes. Infiltration of resin was carried out using the following steps: 1:3 resin to PPO, 1:1 resin to PPO, 3:1 resin to PPO, for 1 hour per each exchange, then 100% resin for 2-3 hours at RT. The resin was then exchanged twice over 1 hour before glands were left to infiltrate for 3 days with 2-3 exchanges of resin per day. Glands were then embedded using two Aclar sheets with a 200 μm gap between them. The Aclar sheet sandwiches were then placed into an oven to polymerise at 60°C for 24 hours. Sections were taken from the polymerised resin blocks at 75 nm thickness using a Leica UC7 microtome and placed onto 200 mesh copper grids (Athene). The salivary glands were then imaged at 11,000 x magnification on a FEI Tecnai electron microscope using a montaging tool (SerialEM).

### Analysis of mitochondrial relocation

Salivary glands and wing imaginal discs from wandering third instar larvae were dissected in ice cold PBS and then fixed in 4% PFA for 30 min (RT). Tissues were permeabilised (4x30 min in PBT-0.3% Triton X-100 for glands; 3x20 min in PBT-0.3% Triton X-100 for discs) and blocked (4x30 min in PBT-0.1% Triton X-100, 10% BSA for glands; 3x20 min in PBT-0.3% Triton X-100, 5% BSA for discs) at RT. Tissues were incubated overnight at 4°C in primary antibodies diluted in 0.1% Triton X-100, 10% BSA or PBT-0.3% Triton X-100, 5% BSA for glands or discs, respectively. Tissues were then washed at RT (4x30min in PBT-0.1% Triton X-100 for glands; 3 x 20min in PBT-0.1% Triton X-100 for discs), before overnight incubation at 4°C in secondary antibodies diluted in 0.1% Triton X-100, 10% BSA or PBT-0.3% Triton X-100, 5% BSA for glands or discs, respectively. Tissues were then washed at RT (4x30min in PBT-0.1% Triton X-100 for glands; 3x20min in PBT-0.1% Triton X-100 for discs) and equilibrated in Vectashield containing DAPI (Vector Laboratories) overnight at 4°C. Tissues were mounted in a fresh drop of the same mounting medium before imaging. Primary antibodies used: mouse anti-Golgin-84 (clone 12-1, 1:50[42]), goat anti-GMAP (1;1000[42]), goat anti-Golgin-245 (1:500-1:1000[42]), Mouse anti-V5 (1:1000, ThermoFisher Scientific), rabbit anti-V5 (1:1000, Cell Signalling Technology), rabbit anti-Syntaxin 16 (1:500, Abcam), rabbit anti-GM130 (1:1000, Abcam), guinea pig anti-GCC88 (1:200[67]), chicken anti-V5 (1:1000, Bethyl), rabbit anti-Lava lamp (1:5000[45]), mouse anti-Complex V α (1:500, ThermoFisher Scientific), rat anti-Glg1 (1-50-1:200[44]), chicken anti-Arl1 (1:200[69]), rabbit anti-Sec16 (1:2000[68]). Other reagents: GFP booster-Atto647N or Atto488 (1:400, Chromotek). Fluorescent secondary antibodies used: donkey anti-mouse 488 (1:1000, ThermoFisher Scientific), donkey anti-rat 488 (1:1000, ThermoFisher Scientific), donkey anti-rabbit 555 (1:1000, ThermoFisher Scientific), donkey anti-chicken Cy3 (1:2000, Jackson ImmunoResearch), donkey anti-goat 555 (1:1000, ThermoFisher Scientific), donkey anti-chicken Cy5 (1:2000, Jackson ImmunoResearch), donkey anti-rabbit 647 (1:1000, ThermoFisher Scientific), donkey anti-mouse 647 (1:1000, ThermoFisher Scientific), donkey anti-rat 647 (1:1000, ThermoFisher Scientific).

Confocal imaging of cargo relocated to mitochondria was performed at Nyquist sampling rate on an upright Leica SP8 confocal microscope using a 63X Apochromat oil-immersion objective (NA = 1.4). For salivary glands, the proximal cells were imaged within a 184.59 x 184.59 μm field of view, proximal cells being defined as those comprising approximately the first third of the salivary gland along the proximo-distal axis and which lack mature secretory granules.[77] For YFP::Pgant9 wing disc mito-relocation experiments, notum cells positive for YFP::Pgant9 and V5 expression were imaged within a 73.88 x 73.88 μm field of view. In all other wing disc mito-relocation experiments, a 131.87 x 65.95 μm image of the wing pouch was taken with the dorso-ventral boundary aligned at the centre of the vertical axis and with posterior compartment cells comprising approximately half of the field of the view. Within each mito-relocation experiment, all samples across all genotypes were imaged with the same settings for the cargo channel.

### Fluorescence imaging of testes

Wandering third instar larvae or 3-5 days old adult males were collected and salivary gland (SG) or male reproductive systems (RS) were dissected in PBS. Tissues were fixed in 4% PFA for 30min and then permeabilised for 4x30min in PBT-0.3% Triton-X100. Tissues were then blocked for 4x30min in PBT-0.1% Triton X-100, 5% BSA and primary antibodies were incubated in PBT-0.1% Triton X-100, 5% BSA o/n at 4°C. Tissues were washed 4x30min in PBT-0.1% Triton-X100 and secondary antibodies (including GFP booster) were incubated in PBT-0.1% Triton X-100, 5% BSA o/n at 4°C. Tissues were washed 4x30min in PBT-0.1% Triton-X100 and equilibrated in Vectashield containing DAPI o/n at -20°C. Tissues were mounted in Vectashield containing DAPI, and imaged on a Zeiss 710 confocal. Golgi polarity, was measured using line profiles across Golgi stacks using Fiji.[73] The length of the lines was kept the same for the same genotype. 10 line profiles were measured for each genotype, and data processed with Prism 8.0 (GraphPad). To measure Golgi size from GM130 confocal micrographs, lines were drawn across the widest point to measure the width of the stack. 5-7 images were taken from 3-4 larvae per genotype, and least 100 Golgi were measured per image. The experiment was repeated four times (three times for *TMF;; golgin-84*). For localisation of Skny-GFP and GFP-LAMP spermatids, fixed and permeabilised tissues were incubated for 1h PBT plus rhodamine phalloidin (1:400, Invitrogen). For phalloidin staining of testes to visualise F-actin fixed and permeabilised tissues were incubated in rhodamine phalloidin (1:400, Invitrogen) for 1.5 h at RT. Tissues were washed in PBT 0.1% Triton X-100 and equilibrated in Vectashield containing DAPI o/n at -20°C. Tissues were mounted in Vectashield containing DAPI and imaged on a Zeiss 710 confocal followed by processing in Fiji. For VVA staining, L3 salivary glands were fixed for 30 min in 4% PFA at RT, and labelled with VVA conjugated with fluorescein (1 μg/ml, Vector Laboratories). Competing sugar,

N-Acetyl-D-Galactosamine (GalNAc, 0.3 M, Sigma). Two technical repeats were done, and six salivary glands from different larvae were analysed for each genotype. Images were taken on a Zeiss 710 confocal microscope and processed in Fiji.

To analyse *TMF, GMAP* and *TMF, GMAP;; golgin-84* mutant testes, the desired mutant pupae/flies were collected from vials and imaged on a $CO_2$ pad using a Leica IC90 E digital camera attached to a Leica M80 stereo-microscope. For testes, the desired mutant pupae/flies were collected from vials and the male reproductive system was dissected in PBS. Testes were either immediately imaged, or fixed for 30 min in 4% PFA at RT prior to imaging on a Leica M80 stereo-microscope.

### CRISPR/Cas9-directed tagging and knockouts

For TMF, EGFP was inserted into a region that is poorly conserved, downstream of the predicted N-terminal vesicle capturing motif and upstream of the first predicted coiled-coil domain. We used the CRISPR Optimal Target Finder to choose a gRNA target site, and pCFD3 was used for BbsI-dependent gRNA cloning.[70,74] The EGFP coding sequence with linker sequences on both sides and ~800 bp 5' and 3' homology arms were cloned into pBluescript. The EGFP donor construct and the pCFD3 gRNA plasmid were co-injected into TH_attP2 embryos. Single crosses of G0 flies and balancer stocks were set up. For αManII, EGFP was inserted at the C-terminus of *α-Man-IIa* (CG18802, FBgn0011740), with a small linker separating the two elements. EGFP sequence including the N-terminal linker and ~800 bp 5' and 3' homology arms were cloned into pBluescript. gRNAs were cloned into pCFD3, and the EGFP donor construct and the pCFD3 gRNA plasmids were co-injected into CFD2 embryos. Single crosses of G0 flies and balancer stocks were set up. For both genes, G0 flies were removed from vials once crosses were going and genomic DNA isolated using microLYSIS-Plus DNA release buffer (Clent Life Science). PCR was used to screen for EGFP in the germline of G0 flies, and the progeny of the GFP-positive founders used to set up single crosses to establish stable lines. F1 flies were screened for the presence of GFP. We identified four independent GFP::TMF stocks derived from two founders. All stocks express GFP::TMF and are homozygous/hemizygous viable. We identified multiple independent αManII-GFP stocks derived from various founders.

For gene knock-outs, gRNAs were cloned into pCFD3 and injected into either CFD2 (for *golgin-84* mutants) or TH_attP2 (for *TMF* and *GMAP* mutants) depending on the gene's chromosomal location. Two pCFD3 plasmids were co-injected for both *TMF* and *golgin-84* to remove the entire coding sequence. G0 flies were crossed to balancer stocks. The F1 progeny was used to set up single crosses to generate stable lines. Once crosses were going F1 flies were removed from vials and used for diagnostic PCRs and sequencing. gDNA was isolated using microLYSIS Plus (Clent Life Sciences).

### Immunoblotting

To validate *GMAP* and *golgin-84* mutants, five testes were dissected in PBS and immediately transferred to a tube with Schneider's insect medium (Sigma). 4x NuPAGE SDS sample buffer (ThermoFisher Scientific) was added, heated for 10 min at 90°C and loaded onto a 3-8% Tris-acetate gel (ThermoFisher Scientific), and after running, blotted on to a nitrocellulose membrane (Amersham). The membrane was blocked in PBS-T 0.1% Tween 20 and 3% skimmed milk powder and 1% BSA for 1h at RT. Primary antibodies were added for 1h at RT. Membrane was washed and secondary antibodies were added for 45 min at RT. Membrane was washed and blot developed using ECL. For Golgin-84 the membrane was stripped using Restore PLUS Western Blot Stripping Buffer (ThermoFisher Scientific). Primary and secondary antibodies used: mouse anti-Golgin-84 (1:100), goat anti-GMAP (1;2000), rabbit anti-βactin (1:2000-1:4000, Abcam), sheep anti-mouse HRP (1:5000, Amersham), donkey anti-rabbit HRP (1:5000, Amersham), mouse anti-goat HRP (1:5000, Santa Cruz Biotechnology).

To examine Sgs3, salivary glands were dissected from wandering third instar larvae in PBS. SG were immediately transferred into 50 μl of RIPA buffer (Sigma) plus protease inhibitors. Ten SG were dissected for each genotype. SG were homogenised and left on ice for 25 min. 15 μl of the lysate was used per gel. NuPAGE SDS sample buffer (ThermoFisher Scientific) with 5% β-mercaptoethanol was added and samples were boiled for 10 min at 90°C, separated on a 4-12% Bis-Tris gel (ThermoFisher Scientific), and transferred to nitrocellulose membrane. The membrane was blocked in PBS-T 0.1% Tween 20 and 1% BSA for 1 hour at RT, and Rabbit anti-Sgs3 (1:200[53]) added o/n at 4°C. Membrane was washed, HRP-labelled anti-rabbit for 1h at RT, and after washing, the blot analysed using a ChemiDoc (BioRad).

## QUANTIFICATION AND STATISTICAL ANALYSIS

Quantification of relocation of Golgi residents to mitochondria was automated using NIS Elements AR software (5.02.03, Nikon). Images of salivary glands from 4-5 different larvae or 5 different wing discs were filtered to remove background and noise for each channel. DAPI signal was used to segment individual cells from each gland or disc, and binaries of cargo/golgin-mito/Golgi or mitochondria/golgin-mito/Golgi were produced by thresholding for intensity level and/or size filtering. Areas of each binary were automatically generated for each cell per gland or disc, with area used rather than intensity as it is less sensitive to variation between samples,[78] and also, relocated vesicles are likely to be arranged in a layer on the mitochondria. The degree of cargo or golgin-mito mitochondrial relocation was quantified in a cell-by-cell manner by calculating either (1) the ratio between the area of cargo/golgin-mito in mitochondria (CM) and area of total cargo/golgin-mito (CT) or (2) the ratio between area of cargo/golgin-mito in mitochondria (CM) and area of total mitochondria (MT). CM/CT was the default measure for all experiments, except for the following: (1) in the experiment shown in Figure 1C andS1B, because the negative control *fkh> no UAS* does not express any golgin-mito and (2) in wing pouch cells, as these cells possess small cell diameters and thus small

numbers of cargo/golgin-mito objects, rendering discrimination between incidental cargo-mitochondrial overlap and strong cargo relocation difficult. The alternative measure CM/MT was used under the assumption that significant relocation would correlate with substantial, rather than marginal, overlap between linked pairs of cargo and mitochondria binary objects. Upon obtaining cell-by-cell CM/CT or CM/MT ratios, these ratios were averaged per gland or disc to obtain larval ratios, and both cell-by-cell and larval ratios were plotted.[79] Statistical analyses were by one-way nested ANOVA using Tukey's multiple comparisons, as described in the Figure Legends, and were performed using Prism (v 8.0, GraphPad Software). All values are in Data S1, and the micrographs used for quantification are deposited in Mendeley Data (Mendeley Data: https://doi.org/10.17632/yr87kr8wbz.1).

**Current Biology, Volume *32***

## Supplemental Information

# *In vivo* characterization of *Drosophila* golgins reveals redundancy and plasticity of vesicle capture at the Golgi apparatus

**Sung Yun Park, Nadine Muschalik, Jessica Chadwick, and Sean Munro**

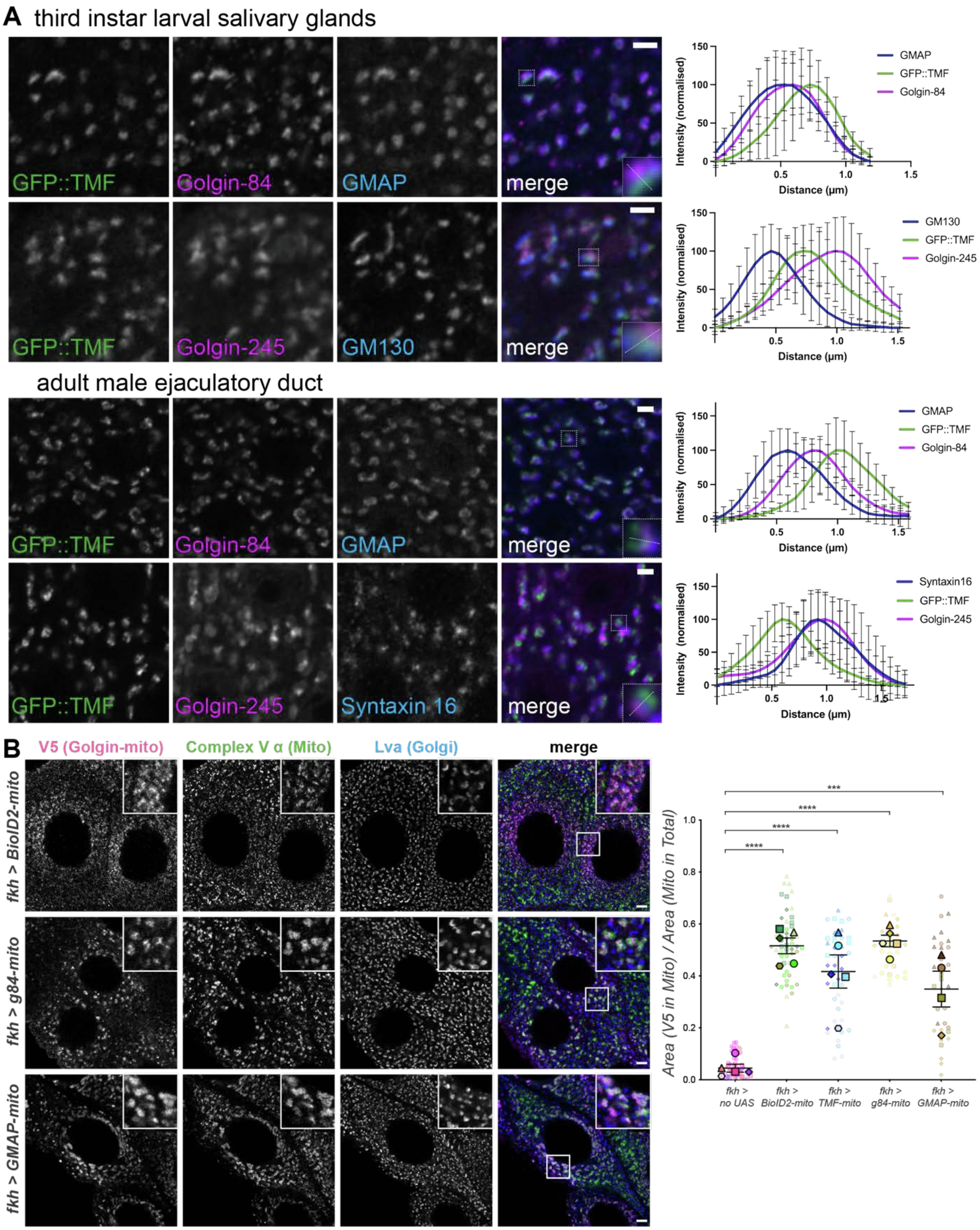


**Figure S1. Localisation of GFP::TMF, and mitochondrial relocation of Golgin-84 and GMAP in *Drosophila*. Related to Figure 1.**

(A) Confocal micrographs comparing GFP::TMF to the indicated golgins in salivary glands and the male ejaculatory duct. The insets show a higher magnification of the Golgi highlighted in the image. The dotted

line illustrates a typical line profile used for quantification, with the graphs showing the normalised means of 10 line profiles of the indicated proteins across the Golgi stack (errors bars, SD). GFP::TMF localises trans to GMAP (cis Golgi) and Golgin-84 (early rims). GFP::TMF localises in between GM130 (cis) and Golgin-245 (trans). GFP::TMF localises cis to Golgin-245 and Syntaxin 16 (both trans Golgi). Scale bars: 2μm. Full data in Data S2.

(B) Confocal micrographs of L3 salivary glands labelled for the V5 epitope tag in the indicated golgin-mito (magenta), and also mitochondrial Complex V α (green) and Golgi-marker Lava lamp (Lva, blue). Insets show zoom-in views of the boxed regions in the merge. Also shown is a negative control (BioID2-mito). Scale bar: 5 μm. Quantification of combined golgin-mito relocation data from Figure 1C and S1A. Degree of relocation calculated by ratio between area of V5 binaries in mitochondria and total area of mitochondria. Cell-by-cell ratios plotted as small partially transparent points. Larval ratios (averaged cell-by-cell ratios) plotted as large opaque points. Points are shaped and coloured according to larval replicate, with the coding scheme of cell-by-cell points matching that of their corresponding larval points. The mean larval ratio (line) and SEM (bars) are plotted for each genotype. Data analysed by one-way nested ANOVA using Tukey's multiple comparisons, *** = $p \le 0.001$, **** = $p \le 0.0001$. n = 5 larvae (4 for GMAP-mito), with 4-15 cells segmented per larvae. Full data in Data S1.

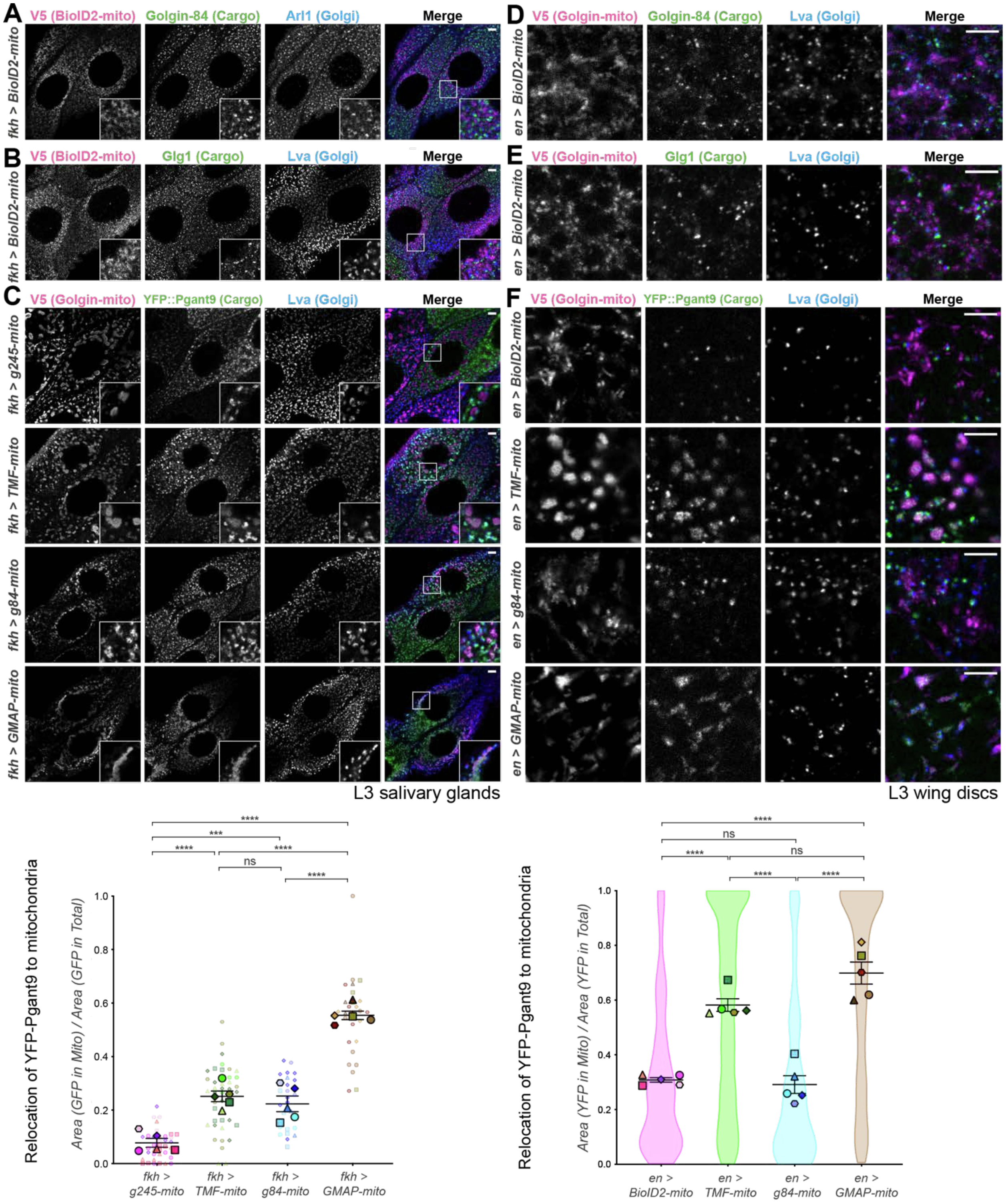


**Figure S2. Capture of YFP::Pgant9 containing vesicles by intra-Golgi golgin-mitos. Related to Figures 2 and 3.**

(A) Confocal micrographs of L3 salivary glands expressing the BioID2-mito negative control, and labelled for V5 (magenta), endogenous Golgin-84 (green), and Golgi-marker Arl1 (blue).

(B) As (A) except the labelling was for V5 (magenta), endogenous Glg1 (green), and *trans*-Golgi marker Lva (blue). Scale bars: 5 μm.

(C) Confocal micrographs of L3 larval salivary glands expressing the indicated V5-tagged golgin-mito constructs and labelled for V5 (magenta), endogenous YFP::Pgant9, and Golgi-marker Lva (blue). Endosome-to-Golgi vesicle tether Golgin-245-mito was used as a negative control. Insets show zoom-in views of the boxed regions in the merge. Quantification of YFP::Pgant9 relocation uses ratios between cargo in mitochondria and total area of cargo. Plots show cell-by-cell ratios plotted as small partially transparent points. Larval ratios (averaged cell-by-cell ratios) plotted as large opaque points. Points are shaped and coloured according to larval replicate, with the coding scheme of cell-by-cell points matching that of their corresponding larval points. The mean larval ratio (line) and SEM (bars) are plotted for each genotype. Data analysed by one-way nested ANOVA using Tukey's multiple comparisons, ns = not significant, ** = $p \leq 0.01$, *** = $p \leq 0.001$, **** = $p \leq 0.0001$. n = 5 larvae for each genotype, with 3-12 Pgant9-positive cells segmented per larvae. Full data in Data S1.

(D) Confocal micrographs of L3 wing discs expressing the BioID2-mito negative control, and labelled for V5 (magenta), endogenous Golgin-84 (green), and Golgi-marker Arl1 (blue).

(E) As (A) except the labelling was for V5 (magenta), endogenous Glg1 (green), and *trans*-Golgi marker Lva (blue). Scale bars: 5 μm.

(F) Confocal micrographs of L3 larval wing discs expressing the indicated golgin-mito constructs and labelled for V5 (magenta), endogenous YFP::Pgant9 (green), and Golgi-marker Lva (blue) in L3 larval wing imaginal disc. YFP::Pgant9 expression was restricted to small patches throughout the wing disc, and so the wing pouch cells imaged for other markers were not imaged, but rather a patch of notum cells, as these cells were one of the few areas where YFP::Pgant9 and *engrailed* expression intersects. Insets show zoom-in views of the boxed regions in the merge. Scale bars: 5 μm. Quantification of YFP::Pgant9 relocation experiment uses the ratio between area of cargo in mitochondria to total area of cargo. For each quantification, cell-by-cell ratios from each larval replicate were pooled for each genotype and represented as truncated violin plots. Disc ratios (averaged cell-by-cell ratios) were plotted as large opaque points. Points are shaped and coloured according to larval replicate, with the coding scheme of cell-by-cell points matching that of their corresponding disc points. The mean larval ratio (line) and SEM (bars) are plotted for each genotype. Data analysed by one-way nested ANOVA using Tukey's multiple comparisons, ns = not significant, * = $p \leq 0.05$, ** = $p \leq 0.01$, *** = $p \leq 0.001$, **** = $p \leq 0.0001$. n = 5 discs for each genotype, with typically 20-55 Pgant9-positive cells segmented per disc. Full data in Data S1.

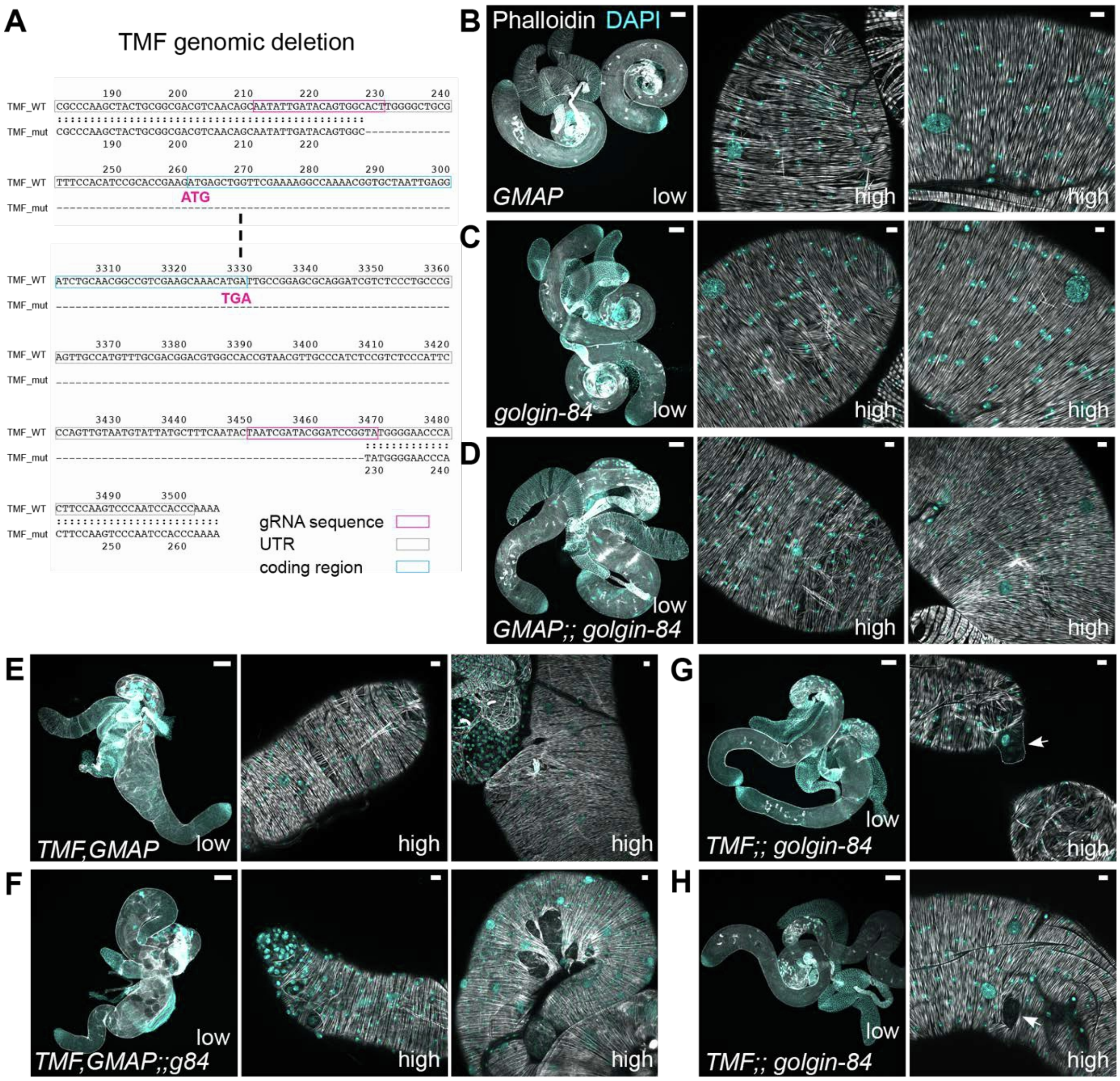


**Figure S3. Testis muscles are not affected in *GMAP* and *golgin-84* single and double mutants. Related to Figures 4 and 5.**

(A) Pairwise sequence alignment of the *TMF* wild-type genomic sequence versus that obtained for a PCR product amplified from the *TMF* deletion mutant. Two gRNAs were used, and this has resulted in the removal of the entire coding region.

(B-H) Confocal micrographs of F-actin (phalloidin, grey scale) and DNA (DAPI, cyan) in testes of the indicated golgin mutants. Images were taken at low magnification to show the whole testes (low), or higher magnification to show the actin in the muscle sheath (high). *GMAP* and *golgin-84* single and double mutants do not show any muscle defects (B-D). Holes (E, F), and lack of muscles at the tip of the testis (F), are the weaker phenotypes observed in *TMF, GMAP* and *TMF, GMAP;; golgin-84* mutants. *TMF;;golgin-84* double mutants (G,H) occasionally lack muscles at the tip of the testis (see dotted line

and arrow in G) and display holes (see arrow in H). The phenotype is similar to that seen in *TMF* single mutants.  Scale bars: low: 100 μm, high: 10 μm.

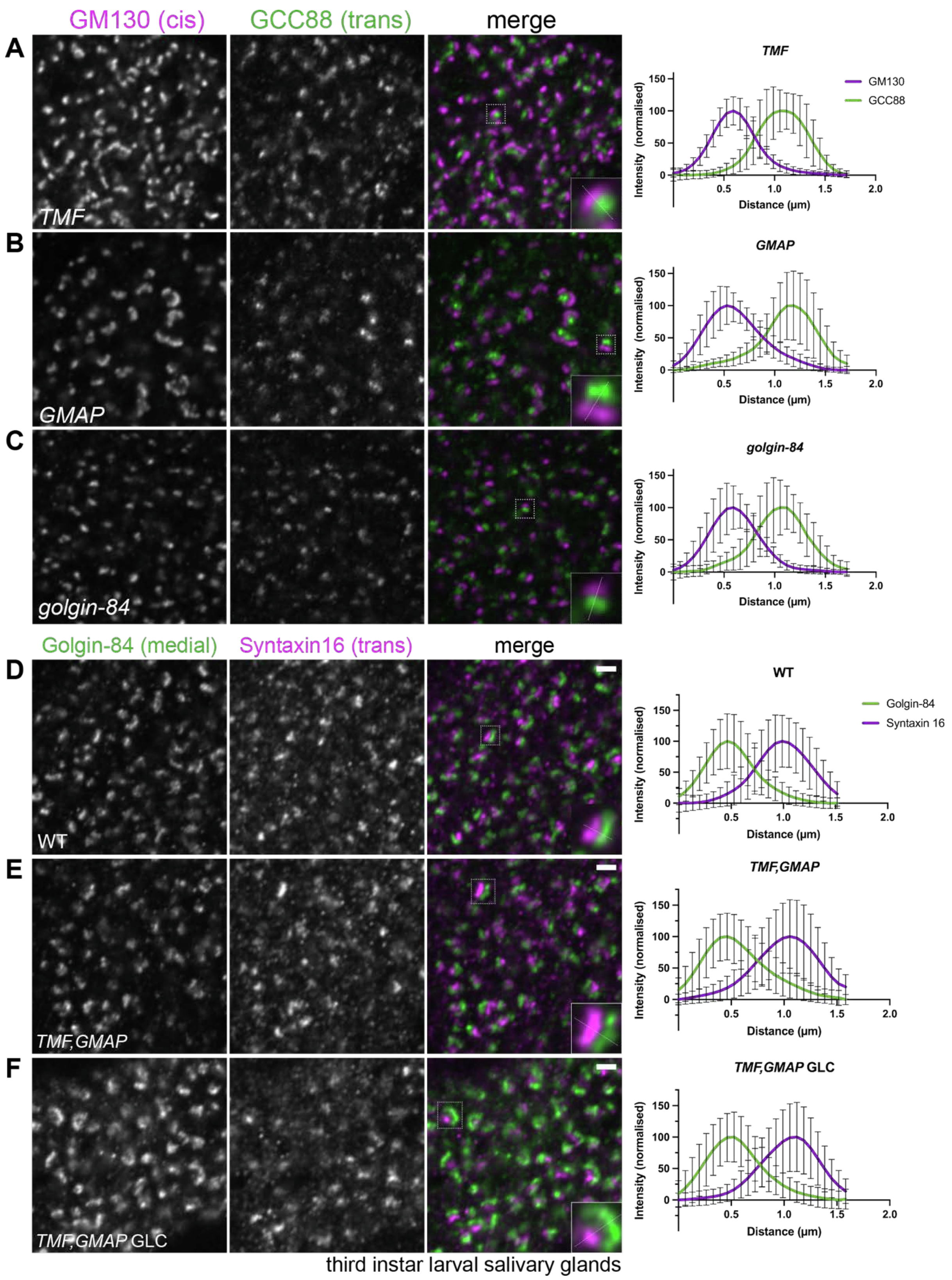


**Figure S4. Intra-Golgi golgin mutants retain Golgi polarity. Related to Figure 6.**

(A-C) Confocal micrographs of L3 salivary gland cells labelled for the cis-Golgi marker GM130 (magenta) and the trans-Golgi marker GCC88 (green). Glands prepared from wild type (WT) or the indicated intra-

Golgi single mutants. The insets show a higher magnification of the Golgi highlighted in the image. The dotted line illustrates a typical line profile used for quantification, with the graphs showing the normalised means of 10 line profiles of GM130 and GCC88 across the Golgi stack. Error bars, SD. Full data in Data S2.

(D-F) Confocal micrographs of L3 salivary gland cells labelled for the medial-Golgi markers Golgin-84 (green)and the trans-Golgi marker Syntaxin16 (magenta). Glands prepared from wild-type (WT), zygotic or zygotic/maternal (germ-line clone, GLC) *TMF,GMAP* double mutants. The germ-line clone ensures that there is no possible maternal contribution of the golgins to the developing progeny, and yet the Golgi is still polarised. The insets show a higher magnification of the Golgi highlighted in the image. The dotted line illustrates a typical line profile used for quantification, with the graphs showing the normalised means of 15-45 line profiles of the marker proteins across the Golgi stack. Error bars, SD. Scale bars: 2 μm. Full data in Data S2.

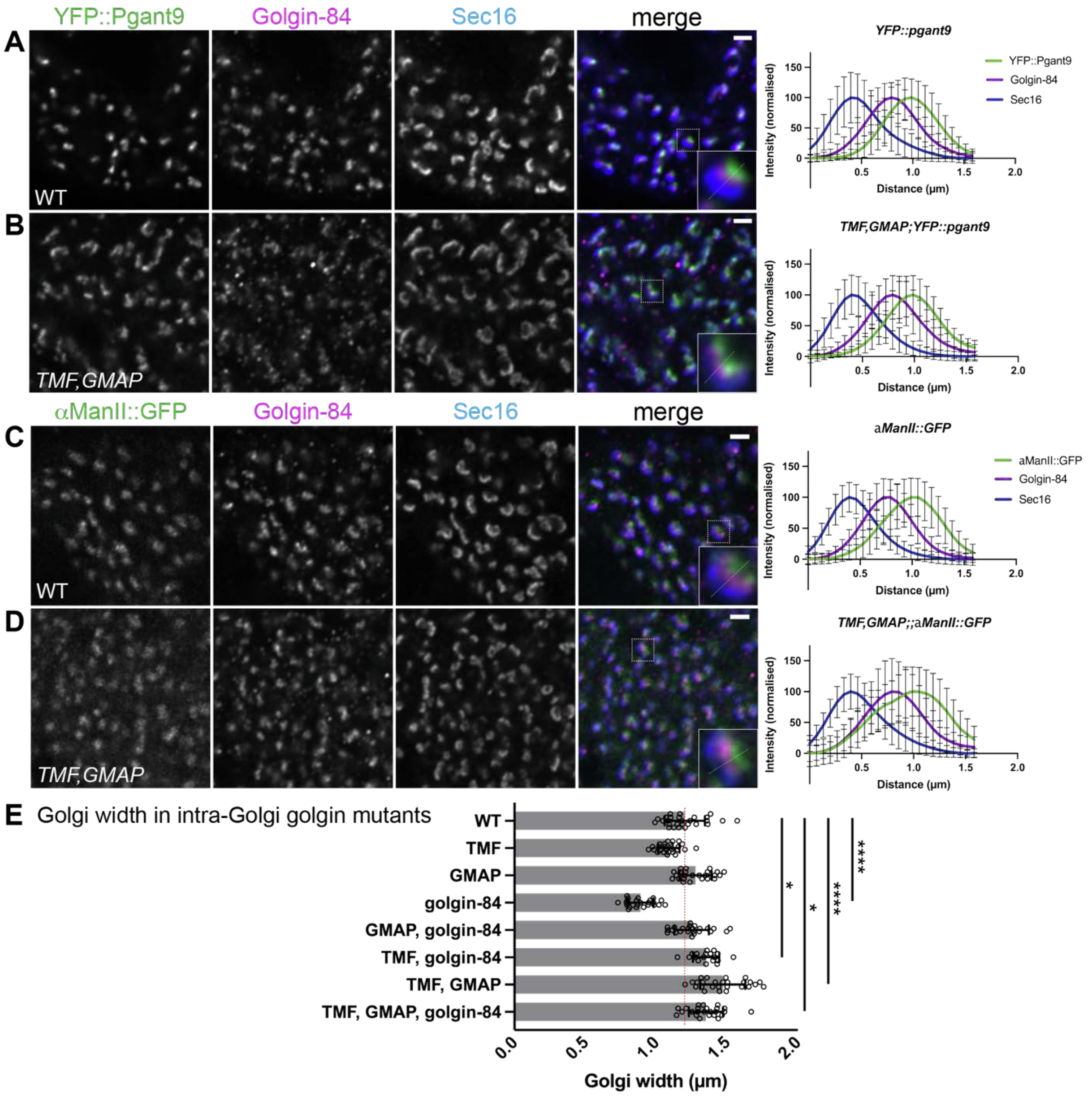


**Figure S5. Characterisation of intra-Golgi golgin mutants. Related to Figure 6.**

(A-D) Confocal micrographs of L3 salivary gland cells labelled for the medial-Golgi markers YFP::Pgant9 (green, A,B) or αManII::GFP (green, C,D) and Golgin-84 (magenta), and the ER exit site marker Sec16 (blue). Glands prepared from *YFP::pgant9* or *αManII::GFP* controls or *TMF,GMAP* double mutants expressing either *YFP::pgant9* or *αManII::GFP*. The insets show a higher magnification of the Golgi highlighted in the image. The dotted line illustrates a typical line profile used for quantification, with the graphs showing the normalised means of 15-45 line profiles of the marker proteins across the Golgi stack. Error bars, SD. Scale bars: 2 μm. Full Data in Data S2.

(E) Quantification of mean Golgi width in intra-Golgi golgin single, double and triple mutants using the GM130 signal for measurements (error bars are SD). 5-7 images from 3-4 larvae were analysed per genotype, and >100 Golgi measured per image. The experiment was repeated four times (three for *TMF;;*

*golgin-84* double mutants). The red dotted line shows the wild type mean for comparison. Statistical significance determined by Kruskal-Wallis using Dunn's multiple comparisons, with significant difference to wild-type indicated: * = $p < 0.05$, **** = $p < 0.0001$. Full data in Data S3.

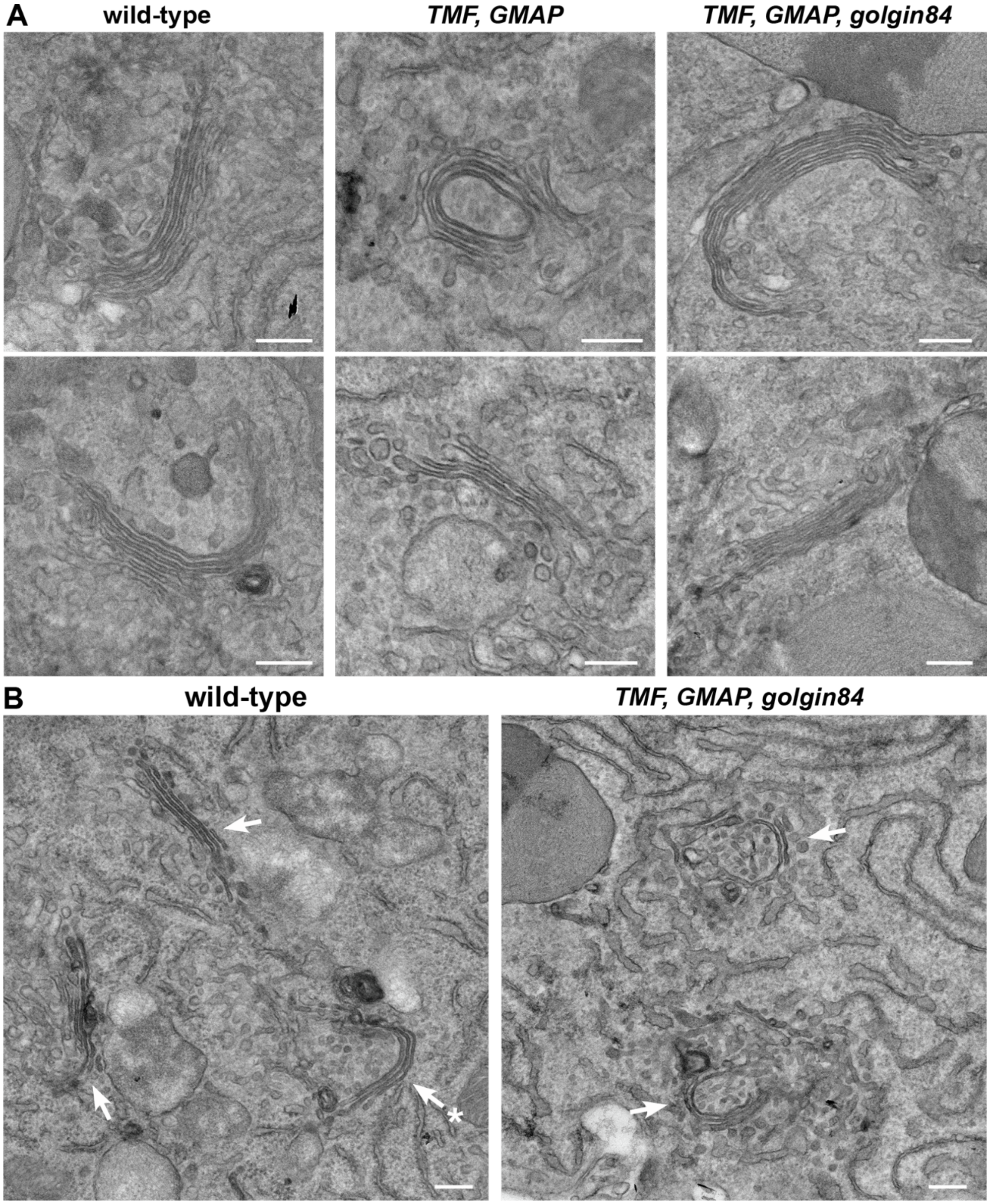


**Figure S6. Golgi stacks are still formed in the absence of the intra-Golgi golgins. Related to Figure 6.**

(A) Golgi stacks from electron micrographs of thin sections of L3 salivary glands from wild type or the indicated mutants. Organised stacks are still present even in the absence of all three intra-Golgi golgins. (B) Electron micrographs as in (A), illustrating the pleiomorphic nature of Golgi stacks (white arrows) in *Drosophila* tissues. The two Golgi stacks in the triple golgin mutant are associated with a large

accumulation of vesicles, but this can also be seen for some stacks in the wild type (asterisk) and so determining its significance will require further investigation by extensive morphological analysis. Scale bars 200 nm.

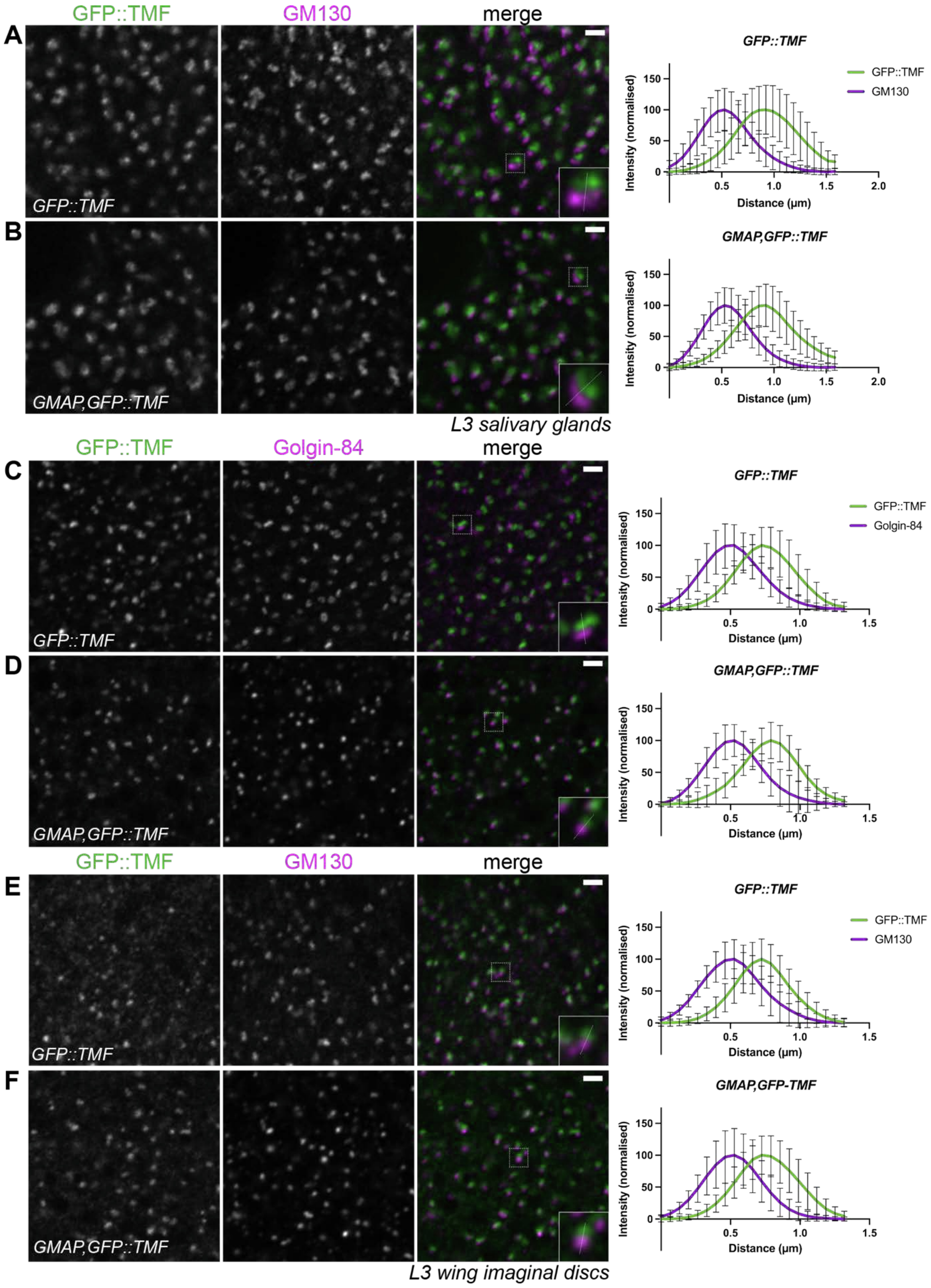


**Figure S7. Loss of GMAP does not affect the localisation of GFP::TMF across the Golgi stack in L3 salivary glands or wing discs. Related to Figure 7.**

(A-B) Confocal micrographs of L3 salivary gland cells labelled for GFP::TMF (green) and the cis-Golgi marker GM130 (magenta). Glands prepared from *GFP::TMF* control or *GMAP,GFP::TMF* mutants. The insets show a higher magnification of the Golgi highlighted in the image. The dotted line illustrates a typical line profile used for quantification, with the graphs showing the normalised means of 15-30 line profiles of the marker proteins across the Golgi stack. Error bars, SD. Scale bars: 2 µm. Full data in Data S2.

(C-F) Confocal micrographs of L3 wing disc cells labelled for GFP::TMF (green) and the medial-Golgi marker Golgin-84 (magenta, C-D) or the cis-Golgi marker GM130 (magenta, E-F). Glands prepared from *GFP::TMF* control or *GMAP,GFP::TMF* mutants. The insets show a higher magnification of the Golgi highlighted in the image. The dotted line illustrates a typical line profile used for quantification, with the graphs showing the normalised means of 15 line profiles of the marker proteins across the Golgi stack. Error bars, SD. Scale bars: 2 µm. Full data in Data S2.
